# Multi-ancestry study of the genetics of problematic alcohol use in over 1 million individuals

**DOI:** 10.1038/s41591-023-02653-5

**Published:** 2023-12-07

**Authors:** Hang Zhou, Rachel L. Kember, Joseph D. Deak, Heng Xu, Sylvanus Toikumo, Kai Yuan, Penelope A. Lind, Leila Farajzadeh, Lu Wang, Alexander S. Hatoum, Jessica Johnson, Hyunjoon Lee, Travis T. Mallard, Jiayi Xu, Keira J. A. Johnston, Emma C. Johnson, Trine Tollerup Nielsen, Marco Galimberti, Cecilia Dao, Daniel F. Levey, Cassie Overstreet, Enda M. Byrne, Nathan A. Gillespie, Scott Gordon, Ian B. Hickie, John B. Whitfield, Ke Xu, Hongyu Zhao, Laura M. Huckins, Lea K. Davis, Sandra Sanchez-Roige, Pamela A. F. Madden, Andrew C. Heath, Sarah E. Medland, Nicholas G. Martin, Tian Ge, Jordan W. Smoller, David M. Hougaard, Anders D. Børglum, Ditte Demontis, John H. Krystal, J. Michael Gaziano, Howard J. Edenberg, Arpana Agrawal, Million Veteran Program, Hongyu Zhao, Amy C. Justice, Murray B. Stein, Henry R. Kranzler, Joel Gelernter

**Affiliations:** 1 Department of Psychiatry, Yale School of Medicine, New Haven, CT USA; 2 Veterans Affairs Connecticut Healthcare System, West Haven, CT USA; 3 Section of Biomedical Informatics and Data Science, Yale School of Medicine, New Haven, CT USA; 4 Crescenz Veterans Affairs Medical Center, Philadelphia, PA USA; 5 Department of Psychiatry, University of Pennsylvania Perelman School of Medicine, Philadelphia, PA USA; 6 Stanley Center for Psychiatric Research, The Broad Institute of MIT and Harvard, Cambridge, MA USA; 7 Analytic and Translational Genetics Unit, Department of Medicine, Massachusetts General Hospital, Boston, MA USA; 8 Psychiatric Genetics, QIMR Berghofer Medical Research Institute, Brisbane, Queensland Australia; 9 School of Biomedical Sciences, Queensland University of Technology, Brisbane, Queensland Australia; 10 Faculty of Medicine, University of Queensland, Brisbane, Queensland Australia; 11 Department of Biomedicine – Human Genetics, Aarhus University, Aarhus, Denmark; 12 The Lundbeck Foundation Initiative for Integrative Psychiatric Research, iPSYCH, Aarhus, Denmark; 13 Center for Genomics and Personalized Medicine, Aarhus, Denmark; 14 Department of Psychological and Brain Sciences, Washington University in St. Louis, Saint Louis, MO USA; 15 Pamela Sklar Division of Psychiatric Genomics, Department of Psychiatry, Icahn School of Medicine at Mount Sinai, New York, NY USA; 16 Department of Genetics and Genomic Sciences, Icahn School of Medicine at Mount Sinai, New York, NY USA; 17 Psychiatric and Neurodevelopmental Genetics Unit, Center for Genomic Medicine, Massachusetts General Hospital, Boston, MA USA; 18 Department of Psychiatry, Massachusetts General Hospital, Harvard Medical School, Boston, MA USA; 19 Department of Psychiatry, Washington University School of Medicine, Saint Louis, MO USA; 20 Child Health Research Centre, The University of Queensland, Brisbane, Queensland Australia; 21 Institute for Psychiatric and Behavioral Genetics, Department of Psychiatry, Virginia Commonwealth University, Richmond, VA USA; 22 Genetic Epidemiology, QIMR Berghofer Medical Research Institute, Brisbane, Queensland Australia; 23 Brain and Mind Centre, University of Sydney, Camperdown, New South Wales Australia; 24 Department of Biostatistics, Yale School of Public Health, New Haven, CT USA; 25 Department of Genetics, Yale School of Medicine, New Haven, CT USA; 26 Vanderbilt Genetics Institute, Vanderbilt University Medical Center, Nashville, TN USA; 27 Department of Medicine, Division of Medical Genetics, Vanderbilt University Medical Center, Nashville, TN USA; 28 Department of Psychiatry and Behavioral Sciences, Vanderbilt University Medical Center, Nashville, TN USA; 29 Department of Psychiatry, University of California San Diego, La Jolla, CA USA; 30 School of Psychology, University of Queensland, Brisbane, Queensland Australia; 31 Center for Precision Psychiatry, Massachusetts General Hospital, Boston, MA USA; 32 Center for Neonatal Screening, Department for Congenital Disorders, Statens Serum Institut, Copenhagen, Denmark; 33 The Novo Nordisk Foundation Center for Genomic Mechanisms of Disease, Broad Institute of MIT and Harvard, Cambridge, MA USA; 34 Department of Neuroscience, Yale School of Medicine, New Haven, CT USA; 35 National Center for PTSD, US Department of Veterans Affairs, West Haven, CT USA; 36 Department of Psychology, Yale University, New Haven, CT USA; 37 Psychiatry and Behavioral Health Services, Yale–New Haven Hospital, New Haven, CT USA; 38 Massachusetts Veterans Epidemiology and Research Information Center (MAVERIC), Boston Veterans Affairs Healthcare System, Boston, MA USA; 39 Department of Medicine, Divisions of Aging and Preventative Medicine, Brigham and Women’s Hospital, Boston, MA USA; 40 Department of Medicine, Harvard Medical School, Boston, MA USA; 41 Department of Biochemistry and Molecular Biology, Indiana University School of Medicine, Indianapolis, IN USA; 42 Department of Medical and Molecular Genetics, Indiana University School of Medicine, Indianapolis, IN USA; 43 Department of Internal Medicine, Yale School of Medicine, New Haven, CT USA; 44 Center for Interdisciplinary Research on AIDS, Yale School of Public Health, New Haven, CT USA; 45 Psychiatry Service, VA San Diego Healthcare System, San Diego, CA USA; 46 Herbert Wertheim School of Public Health and Human Longevity Science, University of California San Diego, La Jolla, CA USA

**Keywords:** Addiction, Genome-wide association studies

## Abstract

Problematic alcohol use (PAU), a trait that combines alcohol use disorder and alcohol-related problems assessed with a questionnaire, is a leading cause of death and morbidity worldwide. Here we conducted a large cross-ancestry meta-analysis of PAU in 1,079,947 individuals (European, *N* = 903,147; African, *N* = 122,571; Latin American, *N* = 38,962; East Asian, *N* = 13,551; and South Asian, *N* = 1,716 ancestries). We observed a high degree of cross-ancestral similarity in the genetic architecture of PAU and identified 110 independent risk variants in within- and cross-ancestry analyses. Cross-ancestry fine mapping improved the identification of likely causal variants. Prioritizing genes through gene expression and chromatin interaction in brain tissues identified multiple genes associated with PAU. We identified existing medications for potential pharmacological studies by a computational drug repurposing analysis. Cross-ancestry polygenic risk scores showed better performance of association in independent samples than single-ancestry polygenic risk scores. Genetic correlations between PAU and other traits were observed in multiple ancestries, with other substance use traits having the highest correlations. This study advances our knowledge of the genetic etiology of PAU, and these findings may bring possible clinical applicability of genetics insights—together with neuroscience, biology and data science—closer.

## Abstract (web summary)

A multi-ancestry genome-wide association study of problematic alcohol use in one million individuals identified 110 risk variants and shows that multi-ancestry polygenic scores improve risk prediction compared with single-ancestry scores

## Main

Excessive alcohol use and alcohol use disorder (AUD) are leading causes of death and morbidity worldwide. Globally, alcohol use accounts for 2.2% of female deaths and 6.8% of male deaths^1^. AUD is a chronic relapsing disease associated with a host of adverse medical, psychiatric and social consequences^2^. According to the 2021 National Survey on Drug Use and Health, 29.5 million people in the United States aged 12 years and older had a Diagnostic and Statistical Manual of Mental Disorders, Fifth Edition (DSM-5)^3^ diagnosis of AUD in the past year. However, fewer than 8.7% of diagnosed individuals had received any treatment for AUD. In addition to psychosocial treatments, only three medications—disulfiram, naltrexone and acamprosate—are approved by the United States Food and Drug Administration for treating AUD, and another two (topiramate and gabapentin) are recommended for off-label use^4^.

Genetic and environmental factors contribute to AUD risk, with an observed heritability (*h*^2^) of ∼50% (ref. ^5^). Identifying genetic factors could advance efforts to prevent, identify and treat both medical and psychiatric aspects related to alcohol. There has been substantial progress made in genome-wide association studies (GWAS) of AUD and related phenotypes^6–10^, including measures of alcohol consumption^11,12^. A prior GWAS of problematic alcohol use (PAU, *N* = 435,563), a phenotype based on a meta-analysis of highly genetically correlated (genetic correlations (*r*_g_) > 0.7) traits—AUD, alcohol dependence (AD) and alcohol-related problems identified using questions 4–10 of the Alcohol Use Disorders Identification Test–Problem (AUDIT–P) questionnaire)—identified 29 independent risk variants, predominantly in European (EUR) ancestry individuals^9^.

A key finding from recent studies is that both AUD and AUDIT–P differ phenotypically and genetically from typical alcohol consumption^7,10,13^. AUD and AUDIT–P index aspects of excessive alcohol intake and higher risk of which correlate with genetic liability to psychiatric and psychosocial factors (for example, higher risk for major depressive disorder and lower educational attainment (EA)). An item-level study of the AUDIT questionnaire confirmed a two-factor structure at the genetic level, underscoring unique genetic influences on alcohol consumption and alcohol-related problems^14^ and noted that the genetics of drinking frequency were confounded by socioeconomic status. A similar pattern—genetic distinctions between substance use disorder (SUD) versus nondependent use—has also been observed for cannabis use disorder and cannabis use^15^. Furthermore, aggregating across multiple SUDs suggests that problematic and disordered substance use has a unique genetic architecture that, while shared across SUDs, does not overlap fully with nondependent substance use per se^16^.

Notwithstanding prior discovery of multiple genome-wide significant (GWS) loci for PAU, there are major gaps in our understanding of its genetic underpinnings. First, the estimated single-nucleotide polymorphism (SNP)-based heritability (*h*^2^) of AUD and PAU ranges from 5.6% to 10.0%, reflecting substantial ‘missing heritability’. Second, most of the available samples used in human genetic studies—including for AUD—are from individuals of EUR genetic ancestry; lack of ancestral diversity is a major problem both for understanding the genetics of these traits, and for potential applications of these genetic discoveries to global populations. Our previous study in the Million Veteran Program (MVP) analyzed AUD in multiple ancestral groups^10^. However, non-EUR samples (*N* = 72,387) were far smaller than EUR samples (*N* = 202,004), resulting in inadequate statistical power and unbalanced gene discovery across ancestral backgrounds.

In this Article, to improve our understanding of the biology of PAU in multiple populations, we conducted substantially larger ancestry-specific GWAS of PAU followed by a cross-ancestry meta-analysis in 1,079,947 individuals from multiple cohorts. We identified 85 independent risk variants in participants of EUR ancestry and 110 in the within-ancestry and cross-ancestry meta-analyses. We investigated the shared genetic architectures of PAU across different ancestries and performed fine mapping for causal variants by combining information from multiple ancestries. We identified dozens of genes linked to brain with convergent evidence. A drug repurposing analysis identified potential medications that have the potential to inform further pharmacological studies. Overall, these findings substantially augment the number of loci that contribute to the risk of PAU, which increases our power to investigate the causal relationships of PAU with other diseases, demonstrating similarity in the genetic architecture across ancestries and helps identify potential druggable targets whose therapeutic potential requires empirical evaluation.

## Results

### Ancestrally diverse data collection

**Fig. 1:**
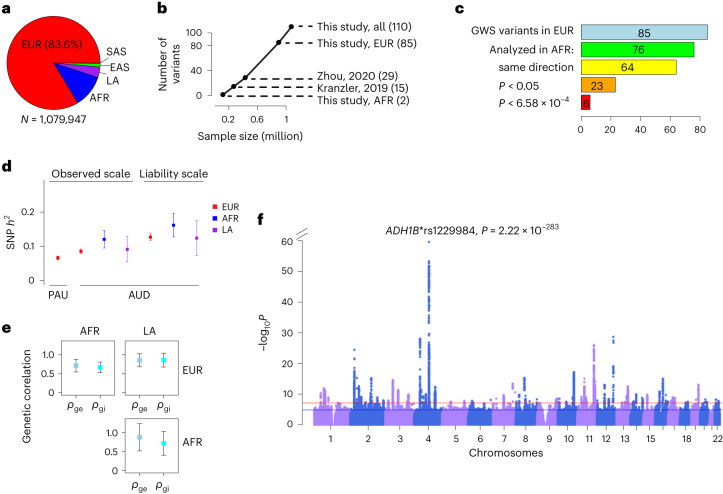
Genetic architecture of PAU. **a**, Sample sizes in different ancestral groups. **b**, Relationship between sample size and number of independent variants identified. Kranzler et al., 2019: cross-ancestry meta-analysis for AUD; Zhou et al., 2020: PAU in EUR. **c**, Lookup for cross-ancestry replication in AFR for the 85 independent variants in the EUR meta-analysis. Of the 85 variants, 76 could be analyzed in AFR (Methods). A sign test was performed for the number of variants with same direction of effect (64/76, binomial test *P* = 1.0 × 10^−9^). Twenty-three variants were nominally significant (*P* < 0.05) in AFR and six were significant after multiple correction (*P* < 0.05/76 = 6.58 × 10^−4^). **d**, Observed-scale and liability-scale SNP-based heritability (*h*^2^) in multiple ancestries. For PAU in EUR, *N* = 903,147 and for AUD, *N* = 753,249 (EUR), *N* = 122,571 (AFR) and *N* = 38,962 (LA). The error bar is the 95% confidence interval. **e**, Cross-ancestry genetic-effect correlation (*ρ*_ge_) and genetic-impact correlation (*ρ*_gi_) among EUR (*N* = 903,147), AFR (*N* = 122,571) and LA (*N* = 38,962) ancestries. The error bar is the 95% confidence interval. **f**, Genome-wide association results for PAU in the cross-ancestry meta-analysis (*N* = 1,079,947 and *N*_effective_ = 646,371). Effective sample size-weighted meta-analyses were performed using METAL. Red line is significance threshold of 5 × 10^−8^.

To extend our understanding of the genetics of PAU—a phenotype comprising AUD and alcohol-related problems measured by the AUDIT–P—we collected data from newly genotyped individuals (most from the MVP^17,18^) and previously published data from multiple cohorts (MVP, FinnGen^19^ and UK Biobank (UKB)^20^, the only cohort that includes AUDIT–P data), the Psychiatric Genomics Consortium (PGC)^8^, iPSYCH^21,22^, the QIMR Berghofer Medical Research Institute (QIMR Berghofer) cohorts^23–25^, Yale–Penn 3 and East Asian (EAS) cohorts (a study of the genetics of methamphetamine dependence in Thailand (Thai METH), Han Chinese–Illumina Global Screening Array (GSA) and Han Chinese–Illumina Cyto12 array (Cyto))^26^) resulting in a total of 1,079,947 individuals (Table 1). Five ancestral groups were analyzed (Fig. 1a): EUR (*N* = 903,147), African (AFR, *N* = 122,571), Latin American (LA, *N* = 38,962), EAS (*N* = 13,551) and South Asian (SAS, *N* = 1,716). As in our previous study^9^, we utilized data on International Classification of Diseases (ICD)-diagnosed AUD (*N*_case_ = 136,182 and *N*_control_ = 692,594), DSM-IV AD (*N*_case_ = 29,770 and *N*_control_ = 70,282) and AUDIT–P (*N* = 151,119), together defined as PAU (based on high genetic correlations (*r*_g_ > 0.7) across these measures). The total number of AUD and AD cases was 165,952, almost double the 85,391 cases in the previously largest study^27^.

**Table 1:** Demographics for cohorts in the meta-analysis of PAU

Cohorts	Traits	*N*_case_	*N*_control_	*N*_total_	*N*_female_ (%)	*N*_effective_	Ref. ^a^
**EUR ancestry**							
MVP	AUD	80,028	368,113	448,141	33,345 (7.4)	262,947	^9^ and new
FinnGen	AUD	8,866	209,926	218,792	123,579 (56.5)	34,027	New^b^
UKB–EUR1	AUDIT–P	–	–	132,001	74,113 (56.1)	132,001	^9^ and new
UKB–EUR2	AUDIT–P	–	–	17,898	10,529 (58.5)	17,898	New
PGC	AD	9,938	30,992	40,930	20,933 (51.1)	23,075	^8^^d^
QIMR AGDS	AD	6,726	4,467	11,193	8,605 (76.9)	10,737	New
QIMR TWINS	AD	2,772	5,630	8,402	4,922 (58.6)	7,430	^8^ and new
QIMR GBP	AD	1,287	751	2,038	1,435 (70.4)	1,897	New
iPSYCH1	AD	2,117	13,238	15,355	8,077 (52.6)	7,301	New
iPSYCH2	AD	1,024	5,732	6,756	3,607 (53.4)	3,475	New
YP3	AD	567	1,074	1,641	854 (52.0)	1,484	New
**Subtotal**	**PAU**	**113,325**	**639,923**	**903,147**	**289,999 (32.1)**	**502,272**	
**AFR ancestry**							
MVP	AUD	36,330	79,100	115,430	16,084 (13.9)	99,583	^10^ and new
PGC	AD	3,335	2,945	6,280	3,124 (49.7)	4,991	^8^
YP3	AD	451	410	861	430 (50.0)	959	New
**Subtotal**	**AUD**	**40,116**	**82,455**	**122,571**	**19,638 (16.0)**	**105,433**	
**LA**							
**MVP**	**AUD**	**10,150**	**28,812**	**38,962**	**3,731 (9.6)**	**30,023**	^10^ and new
**EAS**^**a**^ **ancestry**							
MVP	AUD	701	6,254	6,955	747 (10.7)	2,521	^26^
Han Chinese–GSA	AD	533	2,848	3,381	1,012 (29.9)	1,796	
Thai METH–MEGA	AD	794	1,576	2,370	1,008 (42.5)	2,112	
Thai METH–GSA	AD	127	405	532	263 (49.4)	387	
Han Chinese–Cyto	AD	99	214	313	0 (0)	271	
**Subtotal**	**AUD**	**2,254**	**11,297**	**13,551**	**3,030 (22.4)**	**7,087**	
**SAS ancestry**							
MVP	AUD	107	389	496	67 (13.5)	336	^10^ and new
UKB–SAS	AUDIT–P	–	–	1,220	535 (43.9)	1,220	New
**Subtotal**	**PAU**	**107**	**389**	**1,716**	**602 (35.1)**	**1,556**	
**Total**	**PAU**	**165,952**	**762,876**	**1,079,947**	**317,000 (29.4)**	**646,371**	

Note:

^a^Data either published in previous alcohol GWAS or newly included for this project.

^b^FinnGen summary statistics were downloaded from FinnGen data freeze v5 (https://r5.finngen.fi/).

^c^Included related individuals from UKB.

^d^Reran the PGC AD GWAS in EUR excluding two Australian cohorts.

Cohorts are described in the Methods. UKB–EUR1: genetically defined EUR ancestry White-British by UKB; UKB–EUR2: genetically defined EUR non-White-British participants (Methods); AGDS, the Australian Genetics of Depression Study; TWINS, the Australian twin family study of AUD; GBP, the Australian Genetics of Bipolar Disorder Study; iPSYCH1, phase 1 of iPSYCH; iPSYCH2, phase 2 of iPSYCH; YP3, Yale–Penn 3; *N*_effective_, effective sample size; MEGA, Illumina Multi-Ethnic Global Array.

### Genome-wide association results for PAU

**Extended Data Fig. 1:**
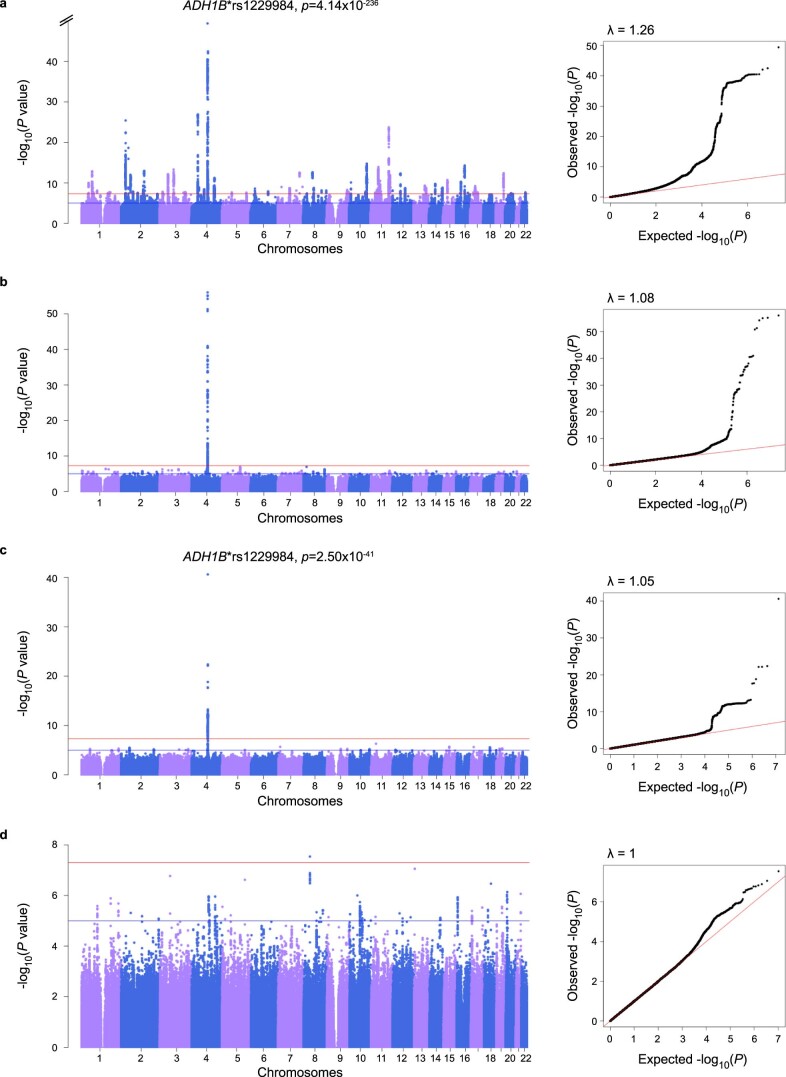
Manhattan and QQ plots for PAU/AUD meta-analyses in different ancestries. **a**, PAU meta-analysis in European ancestry (*N* = 903,147, *N*_effective_ = 502,272). **b**, AUD meta-analysis in African ancestry (*N* = 122,571, *N*_effective_ = 105433). **c**, AUD in Latin Americans (*N* = 38,962, *N*_effective_ = 30,023) from MVP. **d**, PAU meta-analysis in South Asian ancestry (*N* = 1,716, *N*_effective_ = 1,556). Effective sample size-weighted meta-analyses were performed using METAL.

We performed GWAS and within-ancestry meta-analyses for PAU in five ancestral groups and then completed a cross-ancestry meta-analysis. In the EUR meta-analysis, 113,325 cases of AUD/AD, 639,923 controls and 149,899 participants with AUDIT–P scores were analyzed (Extended Data Fig. 1a). After conditional analysis, 85 independent variants at 75 loci reached GWS (Methods, Fig. 1b and Supplementary Table 1). Of these variants, 41 are in protein-coding genes including five missense variants (GCKR*rs1260326, ADH1B*rs75967634, ADH1B*rs1229984, SCL39A8*rs13107325 and BDNF*rs6265).

With the smaller sample numbers, the non-EUR GWAS yielded fewer variants associated with PAU than did the EUR GWAS (Supplementary Table 1). The AFR meta-analysis found two independent *ADH1B* missense variants (rs1229984 and rs2066702) associated with AUD (Fig. 1b and Extended Data Fig. 1b), which have been reported previously^10,28^. In the LA samples from MVP, only ADH1B*rs1229984 (lead SNP) was identified (Extended Data Fig. 1c). Two independent risk variants, ADH1B*rs1229984 and BRAP*rs3782886, were reported in EAS previously^29^. In the small SAS meta-analysis, one intergenic variant (rs12677811) was associated with AUD; however, this SNP was present only in the UKB (Extended Data Fig. 1d).

Of the 85 lead variants identified in the EUR GWAS, 76 were either directly analyzed or had proxy variants in AFR (Methods, Fig. 1c and Supplementary Table 2), 64 of which had the same direction of effect (sign test *P* = 1.00 × 10^−9^). Of these, 23 were nominally associated (*P* < 0.05) and 6 were significantly associated with AUD after multiple-testing correction (*P* < 6.58 × 10^−4^). In LA, 15 of the EUR GWS variants were nominally significant (*P* < 0.05) and 2 were significantly associated with AUD (rs12048727 and rs1229984). In EAS, five variants were nominally significant and two were significantly associated with AUD (rs1229984 and rs10032906). Only two variants were nominally associated with PAU in SAS (rs1229984 was not present in SAS).

The SNP-based heritability (*h*^2^) for PAU and AUD (excluding AUDIT–P from UKB) in EUR, AFR and LA was significant: observed-scale *h*^2^ ranged from 6.6% to 12.7%, and liability-scale *h*^2^ ranged from 12.4% to 16.2% (Fig. 1d and Supplementary Table 3).

**Extended Data Fig. 2:**
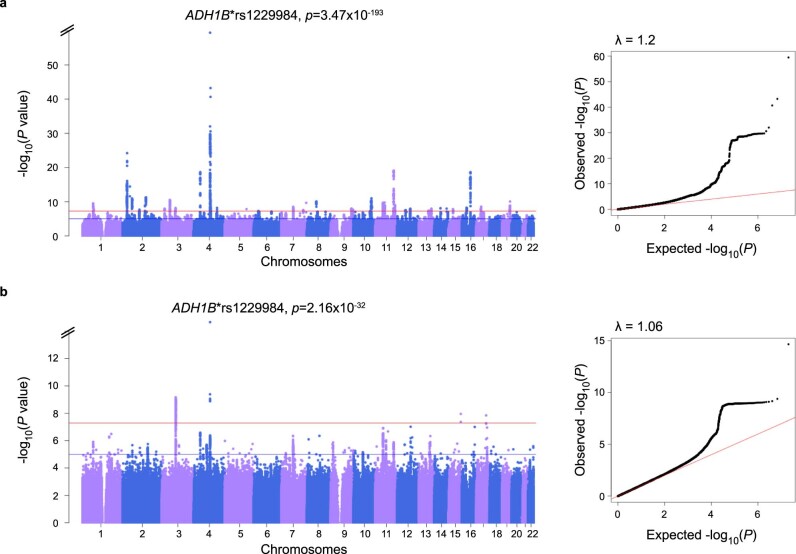
Manhattan and QQ plots for PAU sex-stratified meta-analyses in EUR. **a**, PAU meta-analysis in males (*N* = 496,548, *N*_effective_ = 315,185). **b**, PAU meta-analysis in females (*N* = 143,198, *N*_effective_ = 115,717). Effective sample size-weighted meta-analyses were performed using METAL.

We performed a secondary, sex-stratified (sex was concordant between self-reported and genetically inferred) GWAS in seven EUR samples (Methods). In the analyzed males (*N* = 639,746; Extended Data Fig. 2a), we identified three additional variants associated with PAU: TRIM54*rs142346138 (*P*_males_ = 4.49 × 10^−8^ and *P*_females_ = 0.15), SLC25A48*rs199537352 (*P*_males_ = 1.37 × 10^−8^ and *P*_females_ = 0.98) and CLMN*rs113464470 (*P*_males_ = 9.90 × 10^−9^ and *P*_females_ = 0.38). In females (*N* = 143,198; Extended Data Fig. 2b), we identified two additional variants: intergenic rs72772203 (*P*_females_ = 1.11 × 10^−8^ and *P*_males_ = 0.28) and TLK2*rs181007867 (*P*_females_ = 1.43 × 10^−8^ and *P*_males_ = 0.40). Observed-scale *h*^2^ was estimated to be 8.4% (s.e. 0.3%, *P* = 1.69 × 10^−133^) in males and 4.5% (s.e. 0.5%, *P* = 9.72 × 10^−24^) in females. There was high genetic correlation between males and females (*r*_g_ = 0.84, s.e. 0.04 and *P* = 2.39 × 10^−86^). Overall, we found a similar genetic architecture of PAU in males and females, with possible sex-specific effects at a few loci.

High genetic correlations were observed across the EUR, AFR and LA ancestries (Fig. 1e and Supplementary Table 4). The genetic-effect correlation (*ρ*_ge_) is 0.71 (s.e. 0.09, *P* = 6.16 × 10^−17^) between EUR and AFR, 0.85 (s.e. 0.09, *P* = 3.14 × 10^−22^) between EUR and LA, and 0.88 (s.e. 0.18, *P* = 1.58 × 10^−6^) between AFR and LA. The genetic-impact correlation (*ρ*_gi_) is 0.67 (s.e. 0.07, *P* = 2.78 × 10^−21^) between EUR and AFR, 0.86 (s.e. 0.09, *P* = 3.52 × 10^−20^) between EUR and LA, and 0.72 (s.e. 0.16, *P* = 9.63 × 10^−6^) between AFR and LA. The estimates involving smaller study populations were not robust (Bonferroni *P* > 0.05).

In the cross-ancestry meta-analysis of all available datasets, we identified 100 independent variants at 90 loci (Fig. 1f and Supplementary Table 1); 80 have not been previously reported in association with PAU. Of these, 53 variants were in protein-coding genes, of which 9 are missense variants: GCKR*rs1260326; ADH1B*rs75967634, rs1229984 and rs2066702; SCL39A8*rs13107325; OPRM1*rs1799971; SLC25A37*rs2942194; BDNF*rs6265 and BRAP*rs3782886. The cross-ancestry meta-analysis identified 24 more risk variants than the EUR meta-analysis, but 9 EUR variants fell below GWS (*P* values ranging from 5.26 × 10^−6^ to 9.84 × 10^−8^). In total, 110 unique variants were associated with PAU in either the within-ancestry or cross-ancestry analyses (Fig. 1b and Supplementary Table 1).

### Within- and cross-ancestry causal variant fine mapping

**Fig. 2:**
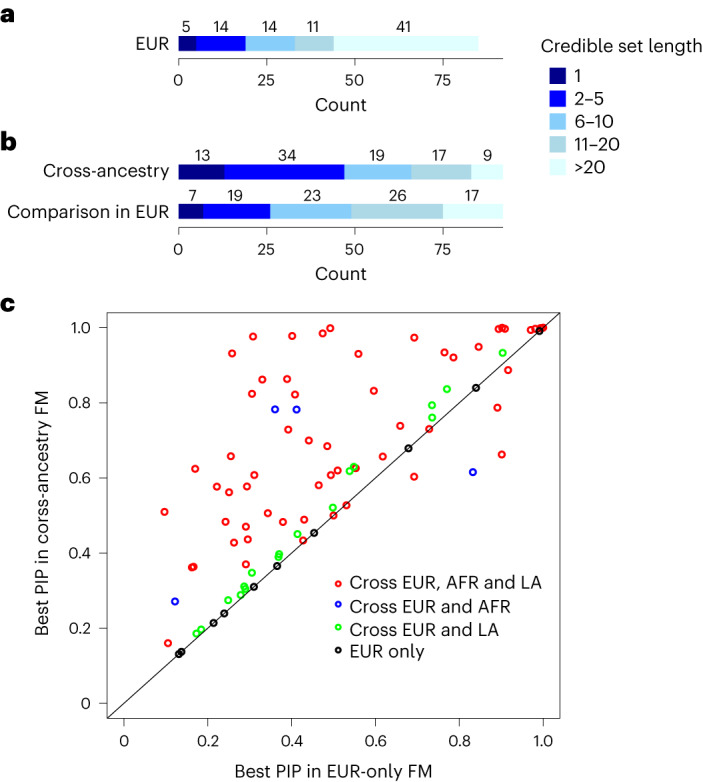
Fine mapping for PAU. **a**, Fine mapping of causal variants in 85 regions in EUR. **b**, Ninety-two regions in a cross-ancestry analysis were fine mapped and a direct comparison was done for these regions in EUR. **c**, Comparison for the highest PIPs from cross-ancestry and EUR-only fine mapping in the 92 regions. Red dots are the regions fine mapped across EUR, AFR and LA; blue dots are the regions fine mapped across EUR and AFR; green dots are the regions fine mapped across EUR and LA; and black dots are the regions only fine mapped in EUR. FM, fine mapping.

We performed within-ancestry fine mapping for the 85 clumped regions with independent lead variants in EUR (Supplementary Tables 5 and 6). A median number of 115 SNPs were included in each region to estimate the credible sets with 99% posterior inclusion probability (PIP) of causal variants. After fine mapping, the median number of SNPs constituting the credible sets was reduced to 20. Among the 85 regions, there were 5 credible sets that include only a single variant with PIP ≥99% (presumably indicating successful identification of specific causal variants): rs1260326 in *GCKR*, rs472140 and rs1229984 in *ADH1B*, rs2699453 (intergenic) and rs2098112 (intergenic). Another 19 credible sets contained ≤5 variants (Fig. 2a).

We performed cross-ancestry fine mapping to identify credible sets with 99% PIP for causal variants proximate to 92 independent lead variants in the cross-ancestry meta-analysis (Supplementary Tables 7 and 8). The median number of SNPs in the credible sets was nine. We found that 13 credible sets contain only a single variant with PIP ≥99%; 47 credible sets contain ≤5 variants (Fig. 2b). For example, fine mapping the region proximate to lead SNP rs12354219 (which maps to *DYPD* on chromosome 1) identified rs7531138 as the most likely potential causal variant (PIP of 48%), although this variant and rs12354219 (PIP of 11%) are in high linkage disequilibrium (LD) in different populations (*r*^2^ ranges from 0.76 to 0.99). In a cross-ancestry meta-analysis, rs7531138-T (the risk allele for PAU) was significantly positively associated with schizophrenia (*P* = 1.04 × 10^−8^), but rs12354219 (*P* = 6.18 × 10^−8^) was not significant^30^. Rs7531138-T was also associated with decreased EA (*P* = 1.74 × 10^−11^), and again, rs12354219 was not (*P* > 5 × 10^−8^)^31^.

To compare within- and cross-ancestry fine mapping, we performed within-ancestry fine mapping for the above 92 regions using the same SNP sets and EUR-only LD information (Fig. 2b,c). The median number of SNPs in the credible sets was 13, with 7 credible sets containing a single variant and 26 containing ≤5 variants, indicating that cross-ancestry fine mapping improved causal variant identification, consistent with other studies reporting improved fine mapping by including other ancestries^12^.

### Gene-based association analysis

We used Multivariate Analysis of Genomic Annotation (MAGMA)^32^ to perform gene-based association analyses. One hundred thirty genes in EUR, nine in AFR and six in LA (for AFR and LA populations, all mapped to the ADH gene cluster), and seven in EAS (mapped to either the ADH gene cluster or the *ALDH2* region) were associated with PAU or AUD (Supplementary Table 9). There were no significant findings in SAS.

### TWAS

We used S-PrediXcan^33^ to identify predicted gene expression associations with PAU in 13 brain tissues. In total, 426 significant gene–tissue associations were identified, representing 89 unique genes (Supplementary Table 10). Five genes showed associations with PAU in all available brain tissues, including aminomethyltransferase (*AMT*), yippee like 3 (*YPEL3*), ecotropic viral integration site 2A (*EVI2A*), ecotropic viral integration site 2B (*EVI2B*) and long noncoding RNA (*CTA-223H9.9*). We also observed associations between PAU and the expression of alcohol dehydrogenase genes (*ADH1B* in the putamen (basal ganglia), *ADH1C* in ten brain tissues and *ADH5* in cerebellar hemisphere and cerebellum). Among the brain tissues, caudate (basal ganglia) had the most genes whose expression was associated with PAU (42 genes), followed by the putamen (basal ganglia, 39 genes). Transcriptome-wide association analyses (TWAS) that integrated evidence across 13 brain tissues using S-MultiXcan^34^ to test joint effects of gene expression variation identified 121 genes (81 shared with S-PrediXcan) whose expression was associated with PAU (Supplementary Table 11).

### Linking risk genes to brain chromatin interaction

We used Hi-C-coupled MAGMA (H-MAGMA)^35^ to implicate risk genes associated with PAU by incorporating brain chromatin interaction profiles. A total of 1,030 gene–chromatin associations were identified in six brain Hi-C annotations, representing 401 unique genes (Supplementary Table 12). Fifty-eight genes showed association with chromatin interaction in all six annotations, including *ADH1B*, *ADH1C*, *DRD2*, *EVI2A* and others that also showed evidence by TWAS in brain tissues.

### Convergent evidence linking association to brain

We examined overlapped genes by both gene-based association analysis and TWAS in brain tissues and/or H-MAGMA analysis using Hi-C brain annotations. Among the 130 genes associated with PAU in EUR, 62 were also implicated by TWAS findings either by single brain tissue (S-PrediXcan) or across brain tissues (S-MultiXcan), 82 have evidence of brain chromatin interaction and 51 have evidence from both TWAS and Hi-C annotations including *ADH1B*, *DRD2*, *KLB* and others (Supplementary Table 9).

### Probabilistic fine mapping of TWAS

We performed fine mapping for TWAS using FOCUS^36^, a method that estimates credible gene sets predicted to include the causal gene, which can be prioritized for functional assays. We detected 53 credible sets at a nominal confidence level (set at 90% PIP). These contained 145 gene–tissue associations with an average PIP of 32% (Supplementary Table 13). For the 19 gene–tissue associations having PIP >90%, 9 are from brain tissues (for example, *ZNF184* expression in the hypothalamus (PIP of 0.94%), *MTCH2* expression in the nucleus accumbens (basal ganglia) (PIP of 99%), *SLC4A8* expression in the dorsolateral prefrontal cortex (PIP of 98%), *YPEL3* expression in the cerebellum (PIP of 100%) and *CHD9* expression in the dorsolateral prefrontal cortex (PIP of 100%).

### Drug repurposing

Independent genetic signals from the cross-ancestry meta-analysis were searched in OpenTargets.org^37^ for druggability and medication target status based on their nearest genes. Among them, *OPRM1* implicated naltrexone and *GABRA4* may implicate acamprosate, both current treatments for AUD. Additionally, *DRD2*, *CACNA1C*, *DPYD*, *PDE4B*, *KLB*, *BRD3*, *NCAM1*, *FTO* and *MAPT* were identified as druggable genes.

From the drug repurposing analysis using S-PrediXcan results, 287 compounds were significantly correlated with the transcriptional pattern associated with risk for PAU (Supplementary Table 14). Of these 287, 141 medications were anticorrelated with the transcriptional pattern. Of those, trichostatin-a (*P* = 3.29 × 10^−35^), melperone (*P* = 6.88 × 10^−11^), triflupromazine (*P* = 7.37 × 10^−10^), spironolactone (*P* = 2.45 × 10^−9^), amlodipine (*P* = 1.42 × 10^−6^) and clomethiazole (*P* = 1.30 × 10^−5^) reversed the transcriptional profile associated with increased PAU risk, targeting a gene near an independent significant locus in the cross-ancestry GWAS.

### Cross-ancestry PRS association

We tested the cross-ancestry polygenic risk score (PRS) association with AUDIT–P in UKB using AUD summary data from EUR (leaving out the UKB AUDIT–P data), AFR and LA. PRS-CSx^38^ was applied to calculate the posterior effect sizes for each SNP by leveraging LD diversity across discovery samples. We validated the PRS associations with AUDIT–P in UKB–EUR2 and tested them in UKB–EUR1 (Table 1). In the UKB–EUR1 samples, the EUR-based AUD PRS was significantly associated with AUDIT–P (*Z* score 11.6, *P* = 3.14 × 10^−31^, covariate-adjusted *R*^2^ = 3.31% and Δ*R*^2^ = 0.11%). By incorporating GWAS data from multiple ancestries, the AUD PRS was more significantly associated with AUDIT–P and explains more variance (Z score 13.6, *P* = 2.44 × 10^−42^, covariate-adjusted *R*^2^ = 3.35% and Δ*R*^2^ = 0.15%) than the single-ancestry AUD PRS.

### Genetic correlations

**Fig. 3:**
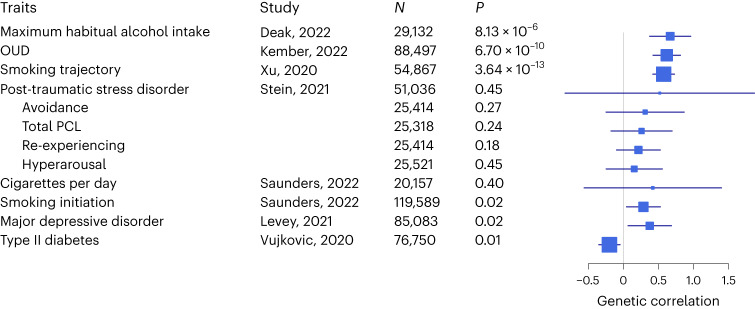
Genetic correlations between AUD and traits in AFR. Total PCL is the total index of recent symptom severity by the post-traumatic stress disorder checklist for DSM-IV. Genetic correlations were estimated using LDSC. Traits with *P* < 3.85 × 10^−3^ are genetically correlated with AUD (*N* = 122,571) after Bonferroni correction. The error bar is the 95% confidence interval.

We confirmed significant positive genetic correlations (*r*_g_) in EUR between PAU and substance use and psychiatric traits (Supplementary Table 15). AD^8^ showed the highest correlation with PAU (*r*_g_ = 0.85, s.e. 0.07 and *P* = 4.49 × 10^−34^), followed by maximum habitual alcohol intake^39^ (*r*_g_ = 0.79, s.e. 0.03 and *P* = 1.24 × 10^−191^) and opioid use disorder (OUD)^40^ (*r*_g_ = 0.78, s.e. 0.04 and *P* = 1.20 × 10^−111^). We next tested *r*_g_ between AUD and 13 published traits with a large GWAS in AFR (Fig. 3 and Supplementary Table 16). Maximum habitual alcohol intake^39^ (*r*_g_ = 0.67, s.e. 0.15 and *P* = 8.13 × 10^−6^) showed the highest correlation with AUD, followed by OUD^40^ (*r*_g_ = 0.62, s.e. 0.10 and *P* = 6.70 × 10^−10^) and smoking trajectory^41^ (*r*_g_ = 0.57, s.e. 0.08 and *P* = 3.64 × 10^−4^).

### PRS for phenome-wide associations

**Extended Data Fig. 3:**
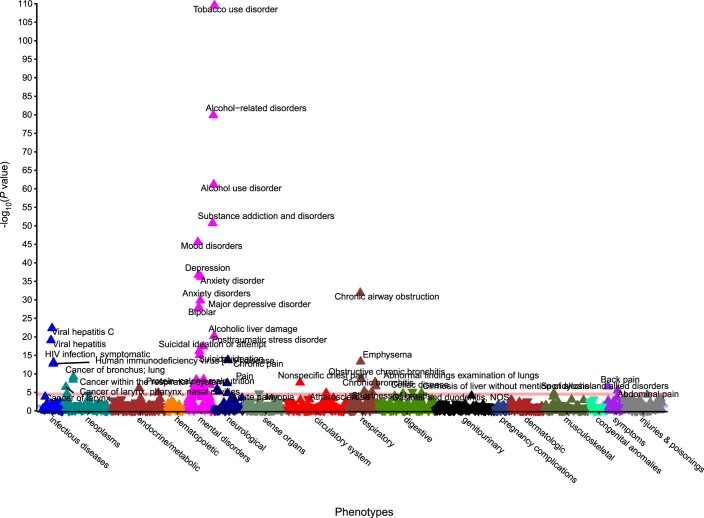
Phenome-wide associations with PAU PRS in PsycheMERGE EUR samples. PheWAS results were meta-analyzed across biobanks (*N* = 131,500). Red line indicates significant after correction for multiple testing (*P* < 0.05/1493 = 3.35 × 10^−5^).

**Extended Data Fig. 4:**
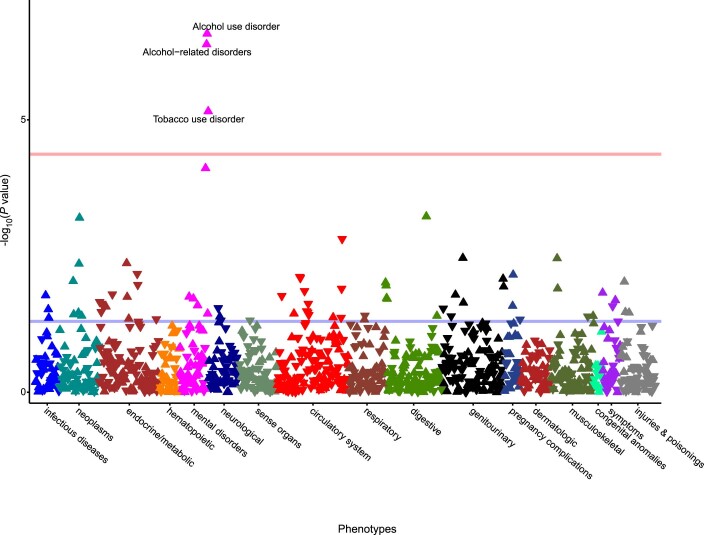
Phenome-wide associations with AUD PRS in PsycheMERGE AFR samples. PheWAS results were meta-analyzed across biobanks (*N* = 27,494). Red line indicates significant after correction for multiple testing (*P* < 0.05/793 = 6.31 × 10^−5^).

In the phenome-wide association studies (PheWAS) using PsycheMERGE data, 58 phenotypes were significantly associated with the PAU PRS in EUR (Supplementary Table 17 and Extended Data Fig. 3). In AFR, AUD (odds ratio (OR) 1.25, s.e. 0.04 and *P* = 2.62 × 10^−7^), alcohol-related disorders (OR 1.21, s.e. 0.04 and *P* = 4.11 × 10^−7^) and tobacco use disorder (OR 1.09, s.e. 0.02 and *P* = 6.98 × 10^−6^) were significantly associated with AUD PRS (Supplementary Table 18 and Extended Data Fig. 4).

**Extended Data Fig. 5:**
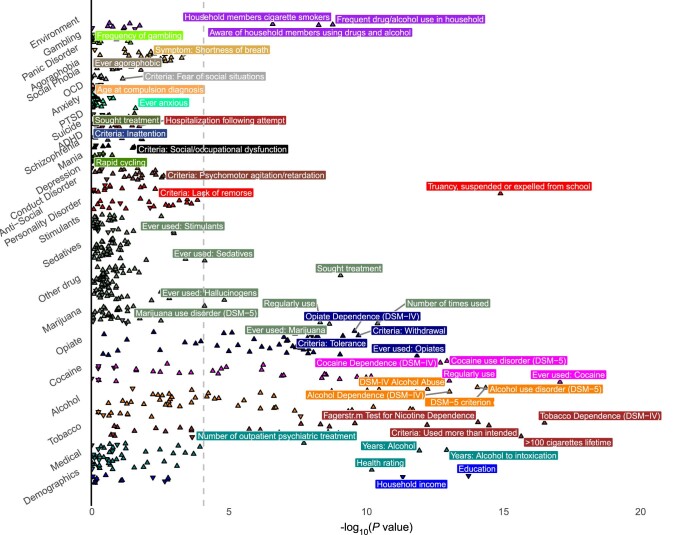
Phenome-wide associations with PAU PRS in Yale-Penn EUR samples. *N* = 5,692. Red line indicates significant after correction for multiple testing (*P* < 0.05/627 = 7.95 × 10^−5^).

**Extended Data Fig. 6:**
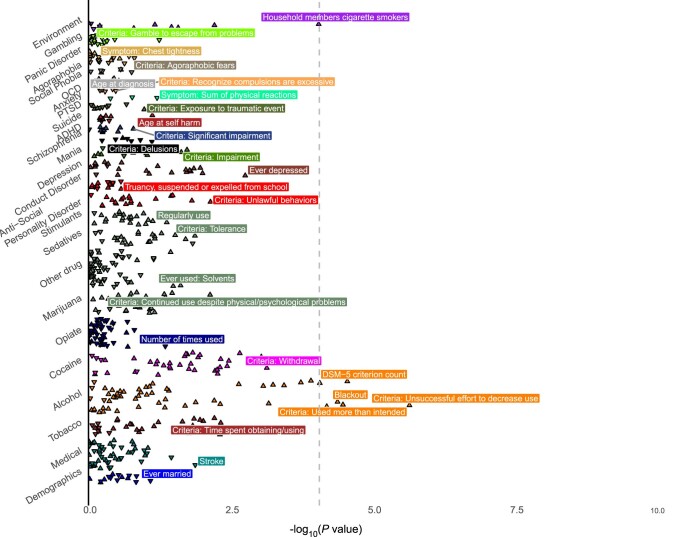
Phenome-wide associations with AUD PRS in Yale-Penn AFR samples. *N* = 4,918. Red line indicates significant after correction for multiple testing (*P* < 0.05/571 = 8.76 × 10^−5^).

In the Yale–Penn EUR subsample, the PRS of PAU was associated with 123 traits, including 26 in alcohol, 39 in opioid, 24 in cocaine and 17 in tobacco categories (Supplementary Table 19 and Extended Data Fig. 5), indicating high comorbidity and shared genetic components across SUDs. In the Yale–Penn AFR subsample, the AUD PRS was associated with six alcohol-related traits, including DSM-5 AUD criterion count, alcohol-induced blackouts and frequency of alcohol use (Supplementary Table 20 and Extended Data Fig. 6).

## Discussion

We report here the largest multi-ancestry GWAS for PAU so far, comprising over 1 million individuals and including 165,952 AUD/AD cases. The inclusion of multiple ancestries both broadened the findings and demonstrated that the genetic architecture of PAU is substantially shared across these populations. Cross-ancestry fine mapping improved the identification of potential causal variants, and cross-ancestry PRS analysis was a better predictor of alcohol-related traits in an independent sample than single-ancestry PRS. We prioritized multiple genes with convergent evidence linking association to PAU with gene expression and chromatin interaction in the brain, and we investigated genetic correlations with multiple traits in AFR, also not possible previously. On the basis of these advances, we identified existing medications predicted to be potential treatments for PAU, which can be tested.

A total of 110 variants were associated with PAU in either within-ancestry or cross-ancestry analyses. These include rs1799971 in *OPRM1* that encodes the *μ* opioid receptor, which plays roles in regulating pain, reward and addictive behaviors. This variant was also associated with OUD on multiple large GWAS^40,42^. Previously, there were inconsistent candidate gene association results for OPRM1*rs1799971 and AUD (reviewed in ref. ^43^). This is the first GWAS to confirm the association of rs1799971 in PAU; the risk allele is the same as for OUD. In contrast to an apparent EUR-specific effect of rs1799971 on OUD, the *OPRM1* association with PAU (*P* = 6.16 × 10^−9^) was detected in the cross-ancestry meta-analysis. Further investigation in larger non-EUR samples is needed to assess the association of this SNP with SUDs in different population groups. Rs6265 in brain-derived neurotrophic factor (*BDNF*) encodes a member of the nerve growth factor family of proteins and has been investigated intensively in the past decades^44^; studies showed that this variant is associated with smoking traits^11^ and externalizing behavior^45^. Rs13107325 in solute carrier family 39 member 8 (*SLC39A8*) has been associated with schizophrenia^46^, substance use^10,11^ and many glycemic traits, and is critical for glycosylation pathways^47^.

The values of liability-scale *h*^2^ of AUD of 12.4% (in LA) to 16.2% (in AFR) can be explained by the current study. Accounting for more of the heritability of a complex trait depends on the genetic architectures of the trait and the power of the study samples. For example, in a whole-genome sequencing study of height, the SNP heritability of height was estimated to be 0.68 (s.e. 0.1), which is close to the pedigree estimates of 0.7–0.8 (ref. ^48^). This is probably due in part to the accuracy with which height is measured and its relative stability once adulthood is reached, and rare variants, in particular those in regions of low LD, that are a major source of the still-missing heritability. A whole-genome sequencing study is warranted to increase our knowledge of the heritability and to identify rare variants contributing to risk for PAU/AUD.

Previous studies have shown that PAU is a brain-related trait with evidence of functional and heritability enrichment in multiple brain regions. We performed gene-based association, TWAS in brain tissues, and H-MAGMA analysis in brain annotations. We identified 51 genes that were supported across multiple levels of analysis. For example, *ADH1B* expression in putamen was associated with PAU by TWAS, and with chromatin interaction in all 6 brain annotations by H-MAGMA, indicating additional potential biological mechanisms for the association of *ADH1B* with PAU risk through gene expression and/or chromatin interactions in brain, potentially independent of the well-known hepatic effect on alcohol metabolism. *DRD2* expression in cerebellar hemisphere and chromatin interaction in all brain annotations were also associated with PAU risk. Alcohol metabolism, as is well reported, has effects that modulate alcohol’s aversive and reinforcing effects^49^, but also contributes to brain histone acetylation, gene expression and alcohol-related associative learning in mice^50^.

In other fields, there has been progress in translating recent knowledge on genetic mechanisms into more effective therapeutic applications^51^. A UKB whole-exome sequencing study identified 564 genes associated with health-related traits, include 36 (6.4%) gene targets of drugs approved by the Food and Drug Administration, which is more common than in the remaining genes (1.9% are gene targets of approved drugs)^52^. Several genes associated with PAU encode proteins that interact with medications approved to treat AUD (for example, *GABRA4* with acamprosate and *OPRM1* with naltrexone^53^). Our multivariate analysis provided evidence for several potentially repurposable drugs. Trichostatin-a, a histone deacetylase inhibitor, showed effects on H3 and H4 acetylation and neuropeptide Y expression in the amygdala, and prevented the development of alcohol withdrawal-related anxiety in rats^54^. Spironolactone, a mineralocorticoid receptor antagonist, reduced alcohol use in both rats and humans in a recent study^55^. Clomethiazole, a GABA receptor antagonist, also showed an effect in treating alcohol withdrawal syndrome^56^. We anticipate that the prioritization of genes in this study will lead to follow-up studies that could improve the likelihood of successful drug development. However, the pathway from genetic variants to the function of encoded protein to a biologically important therapeutic target is complicated and intricate, requiring more work in many modalities.

The PheWAS analyses identified associations with medical phenotypes in EUR. With increasing number of AFR GWAS now published, mainly from MVP, we were able to estimate genetic correlations between AUD and a limited set of traits in AFR. As in EUR, AUD in AFR was genetically correlated with substance use traits including OUD, smoking trajectory (that identifies groups of individuals that follow a similar progression of smoking behavior), and maximum habitual alcohol intake. PheWAS of PRS in AFR from PsycheMERGE and Yale–Penn confirmed that AUD is genetically correlated with substance use traits. The lack of a wider set of phenotypes for comparison by ancestry is a continuing limitation.

Limitations include that the differences in ascertainment and phenotypic heterogeneity across cohorts might bias the results. Despite the high genetic correlation between AUD and AUDIT–P, they are not identical traits, which introduces heterogeneity. Also, differences in ascertainment among the cohorts may have introduced biases; for example, the QIMR Berghofer Australian Genetics of Depression Study (AGDS) cohort has high major depression comorbidity, and the Australian Genetics of Bipolar Disorder Study (GBP) cohort has high bipolar disorder comorbidity. Heterogeneity would, however, have been more likely to limit discovery than to create false positives. Additionally, although we tried to include all available samples for problematic drinking in multiple ancestries, the sample sizes in the non-EUR ancestries were still small for gene discovery and downstream analyses. The collection of data from individuals of diverse genetic ancestries is a critical next step in this field. With more multi-ancestral biobanks and large consortia becoming available, including future releases of data from MVP, the Global Biobank Meta-analysis Initiative^57^ and the All of Us Research Program^58^, we anticipate that the gap between findings in EUR and other populations will diminish. Confounding effects, including socioeconomic status, may bias our results; the *r*_g_ with EA is −0.21 (*P* = 7.57 × 10^−31^), indicating a shared genetic architecture between PAU and EA, a socioeconomic factor that influences many psychiatric traits (and nonpsychiatric traits as well)^31^. Genetic nurture, or indirect genetic effects—effects of alleles in parents on offspring through the environment—exist in many GWAS^59^. Imputation of parental genotypes using family data could improve estimates of direct genetic effects for PAU^60^. We note that the current findings are not sufficient for clinical risk prediction at the individual level, given the limited SNP-based heritability and small proportion of variance explained by PRS.

In summary, we report here a large multi-ancestry GWAS and meta-analysis for PAU, in which we focused our analyses in three main directions. First, we demonstrated that there is substantial shared genetic architecture of PAU across multiple populations. Second, we analyzed gene prioritization for PAU using multiple approaches, including cross-ancestry fine mapping, gene-based association, brain-tissue TWAS and fine mapping, and H-MAGMA for chromatin interaction. We identified many genes associated with PAU with biological support, extending our understanding of the brain biology that substantially modifies PAU risk and expands opportunities for investigation using in vitro methods and animal models. These genes are potential targets for downstream functional studies and studies of potential pharmacological intervention based on the drug repurposing results. Third, we investigated the genetic relationship between PAU and many traits, which was possible in populations of AFR ancestries for the first time.

## Methods

### Ethics

The central Veterans Affairs (VA) institutional review board (IRB) approved the MVP study. All relevant ethical regulations for work with human subjects were followed in the conduct of the study and informed consent was obtained from all participants. The iPSYCH study was approved by the scientific ethics committee in the Central Denmark Region (case no. 1-10-72-287-12) and the Danish Data Protection Agency. The QIMR Berghofer study was approved by the QIMR Berghofer Medical Research Institute Human Research Ethics Committee. The Yale–Penn study was approved by Yale Human Research Protection Program and University of Pennsylvania IRB.

### Study design

In the previous PAU study^9^, the *r*_g_ between MVP AUD and PGC AD was 0.98, which justified the meta-analysis of AUD (includes AUD and AD) across the two datasets, and the *r*_g_ between AUD and UKB AUDIT–P was 0.71, which justified the proxy-phenotype meta-analysis of PAU (including AUD, AD and AUDIT–P) across all datasets. In this study, we use the same definitions, defining AUD by meta-analyzing AUD and AD across all datasets, and defining PAU by meta-analyzing AUD, AD and AUDIT–P (Table 1). No statistical method was used to predetermine sample size.

### MVP dataset

MVP enrollment and genotyping have been described previously^17,18^. MVP is a biobank supported by the United States Department of VA with rich phenotypic data collected using questionnaires and the VA electronic health record system.

MVP genotype data were processed by the MVP release 4 (R4) data team. A total of 729,324 samples were genotyped using an Affymetrix Axiom biobank array. Rigorous sample-level quality control (QC) served to remove samples with duplicates, call rates <98.5%, sex mismatches, >7 relatives or excess heterozygosity. After QC, MVP R4 data contained 658,582 participants and 667,995 variants (pre-imputation). Pre-imputation QC removed variants with high missingness (>1.5%), that were monomorphic, or with Hardy–Weinberg equilibrium (HWE) *P* value of ≤1 × 10^−6^, leaving 590,511 variants for imputation. As in our previous work, we ran a principal component analysis (PCA)^61^ for the R4 data and 1000 Genome phase 3 reference panels^62^. The Euclidean distances between each MVP participant and the centers of the five reference ancestral groups were calculated using the first ten principal components (PCs), with each participant assigned to the nearest reference ancestry. A second round of PCA within each assigned ancestral group was performed and outliers with PC scores >6 standard deviations from the mean of any of the 10 PCs were removed. This two-stage approach resulted in the assignment of 468,869 EUR ancestry, 122,024 AFR, 41,662 LA, 7,364 EAS and 536 SAS individuals for analysis.

Imputation was done by the MVP R4 data team. The entire cohort was prephased using SHAPEIT4 (v4.1.3) (ref. ^63^), then imputed using Minimac4 (ref. ^64^) with the African Genome Resources reference panel by the Sanger Institute and the 1000 Genomes Project phase 3 as reference. Single-nucleotide variants with an imputation score <0.8, HWE *P* value ≤1 × 10^−6^ or minor allele frequency (MAF) lower than the threshold set in each ancestral group based upon their sample size (EUR, 0.0005; AFR, 0.001; LA, 0.005; EAS, 0.01; and SAS, 0.01) were removed before association analysis.

Participants with at least one inpatient or two outpatient ICD-9/10 codes for AUD were assigned as AUD cases, while participants with zero ICD codes for AUD were controls. Those with one outpatient diagnosis were excluded from the analysis. In total, 80,028, 36,330, 10,150, 701 and 107 cases were included in EUR, AFR, LA, EAS and SAS, respectively, and 368,113, 79,100, 28,812, 6,254 and 389 controls were included in EUR, AFR, LA, EAS and SAS, respectively. BOLT-LMM^65^ was used to correct for relatedness, with age, sex and the first ten PCs as covariates.

### UKB

UKB released genotype and imputed data for ∼500,000 individuals from across the United Kingdom^20^, which were accessed through application 41910. UKB defined White-British (WB) participants genetically. For the non-WB individuals, we used a PCA to classify them into different genetic groups, as was performed for MVP. Individuals with available AUDIT–P scores were included in this study. The final sample included 132,001 WB (hereafter called UKB–EUR1) and 17,898 non-WB EURs (hereafter called UKB–EUR2), and 1,220 SAS. SNPs with genotype call rate >0.95, HWE *P* value >1 × 10^−6^, imputation score ≥0.8 and MAF ≥0.001 in EUR1 and EUR2 and ≥0.01 in SAS were kept for GWAS. BOLT-LMM was used for association correcting for relatedness, age, sex and the first ten PCs.

### FinnGen

Summary statistics for AUD from FinnGen data freeze 5 were downloaded from the FinnGen website (http://r5.finngen.fi/). Details of the genotyping, imputation and QC for FinnGen data were described previously^19^. There were 8,866 AUD cases defined by ICD-8/9/10 codes and 209,926 controls. Association analysis was performed using a SAIGE^66^ mixed model with age, sex and ten PCs as covariates. Positions of the variants were lifted over to build 37 (GRCh37/hg19) for meta-analysis.

### iPSYCH

The iPSYCH^21,22^ samples were selected from a baseline birth cohort comprising all singletons born in Denmark between 1 May 1981 and 31 December 2008.

AUD was diagnosed according to the ICD-10 criteria (F10.1–F10.9 diagnosis codes). The iPSYCH cohort was established to investigate genetic risk for major psychiatric disorders (that is, attention-deficit/hyperactivity disorder, schizophrenia, bipolar disorder, major depressive disorder and autism spectrum disorder) but not AUD (or PAU), so comorbidity of psychiatric disorders among these AUD cases is higher than expected for cases selected randomly from the population. Therefore, we generated a control group around five times as large as the case groups and, to correct for the bias introduced by high comorbidity of psychiatric disorders among cases, we included within the control group individuals with the above listed psychiatric disorders (without comorbid AUD) at a proportion equal to what was observed among the cases.

The samples were genotyped in two genotyping rounds referred to as iPSYCH1 and iPSYCH2. iPSYCH1 samples were genotyped using Illumina’s PsychArray and iPSYCH2 samples using Illumina´s GSA v.2 (Illumina). QC and GWAS were performed using the Ricopili pipeline^67^. More details can be found in ref. ^68^. GWAS were performed separately for iPSYCH1 (2,117 cases and 13,238 controls) and iPSYCH2 (1,024 cases and 5,732 controls) using dosages for imputed genotypes and additive logistic regression with the first five PCs (from the final PCAs) as covariates using PLINK v1.9 (ref. ^69^). Only variants with a MAF >0.01 and imputation score >0.8 were included in the final summary statistics.

### QIMR Berghofer cohorts

The AGDS recruited >20,000 participants with major depression between 2017 and 2020. Recruitment and subject characteristics have been reported^23^. Participants completed an online self-report questionnaire. Lifetime AUD was assessed on DSM-5 criteria using the Composite International Diagnostic Interview. A total of 6,726 individuals with and 4,467 without AUD were included in the present study.

The Australian twin family study of AUD (TWINS, including Australian Alcohol and Nicotine Studies) participants were recruited from adult twins and their relatives who had participated in questionnaire- and interview-based studies on alcohol and nicotine use and alcohol-related events or symptoms (as described in ref. ^70^). They were predominantly of EUR ancestry. Young adult twins and their non-twin siblings were participants in the Nineteen and Up study^24^. A total of 2,772 cases and 5,630 controls were defined using DSM-III-R and DSM-IV criteria. Most alcohol-dependent cases were mild, with 70% of those meeting AD criteria reporting only three or four dependence symptoms and ≤5% reporting seven dependence symptoms.

The GBP study recruited >5,000 participants living with bipolar disorder between 2018 and 2021. The sample’s recruitment and characteristics have been reported^25^: participants completed an online self-report questionnaire. Lifetime DSM-5 AUD was assessed using the Composite International Diagnostic Interview.

QIMR cohorts were drawn from larger batches genotyped over an extended period using several different Illumina genotyping microarrays. The microarrays used were (1) Global Screening Array v1 or v2 used for AGDS and GBP, and for TWINS participants either GSA (*N* = 48); (2) Illumina Omni or Core+Exome family chips (Core+Exome *N* = 1,023, PsychArray *N* = 255, OmniExpress *N* = 102 and 2.5M *N* = 321; total *N* = 1,701) or (3) older Illumina HapMap-derived chips (370K *N* = 3,728, 610K *N* = 2,319, 317K *N* = 580 and 660K *N* = 27; total *N* = 6,654). Per-batch imputation QC removed variants with GenTrain score <0.6, MAF <0.01, SNP call rate <95% and HWE deviation (*P* < 1 × 10^−6^). Genotypes from each of the three Illumina microarray families were merged for the core set of markers that passed QC in all batches, then were imputed using the TOPMed Imputation Server with the TOPMed-r2 reference panel^64,71^. The core set used ∼441K, ∼232K and ∼280K markers for (1), (2) and (3), respectively. Association analysis was performed using SAIGE with the LOCO = TRUE flag; age, sex, ten PCs and two covariates that model the three imputation runs, which were used for the individuals. Participants of non-EUR ancestry (defined as >6 standard deviations from the PC1 and PC2 centroids) were excluded. Association analyses were limited to variants with a MAF ≥0.0001, minor allele count ≥5 and an *R*^2^ ≥ 0.1.

### PGC

Lifetime DSM-IV diagnosis of AD in both EUR and AFR ancestries were analyzed by PGC, with details reported previously^8^. This included 5,638 individuals from Australia. To avoid overlap with the new QIMR Berghofer cohorts, we re-analyzed the PGC data without two Australian cohorts: Australian Alcohol and Nicotine Studies and Brisbane Longitudinal Twin Study. This yielded 9,938 cases and 30,992 controls of EUR ancestry and 3,335 cases and 2,945 controls of AFR ancestry.

### Yale–Penn 3

There are three phases of the Yale–Penn study defined by genotyping epoch; the first two were incorporated in the PGC study, thus they are included in the meta-analyses. Here, we included Yale–Penn 3 individuals as a separate sample. Lifetime AD was diagnosed based on DSM-IV criteria. Genotyping was performed in the Gelernter laboratory at Yale using the Illumina Multi-Ethnic Global Array, then imputed using Michigan imputation server with Haplotype Reference Consortium reference. We performed PCA analyses to classify EAs (567 cases and 1,074 controls) and AAs (451 cases and 410 controls). Variants with MAF >0.01, HWE *P* value >1 × 10^−6^ and imputation quality score (INFO) ≥0.8 were retained for association analyses using linear mixed models implemented in GEMMA^72^ and corrected for age, sex and ten PCs.

### EAS cohorts

Summary statistics for AUD/AD GWAS from five EAS cohorts (MVP EAS, Han Chinese–GSA, Thai METH–MEGA, Thai METH–GSA and Han Chinese–Cyto) were included in the cross-ancestry meta-analysis. Analyses of these five cohorts were previously published and the detailed QC can be found in ref. ^26^.

### Meta-analyses

Meta-analyses were performed using METAL^73^ with effective sample size weighting. For all the case-control samples, we calculated effective sample size as:

$${n}_{\mathrm{effective}}=\frac{4}{\frac{1}{{n}_{\mathrm{case}}}+\frac{1}{{n}_{\mathrm{control}}}}$$

For AUDIT–P in UKB, a continuous trait, we used actual sample sizes for meta-analysis. For all meta-analyses within or across ancestries, variants with a heterogeneity test *P* value <5 × 10^−8^ and variants with effective sample size <15% of the total effective sample size were removed. For the cross-ancestry and EUR within-ancestry meta-analyses, we required that variants were present in at least two cohorts. For the AFR and SAS within-ancestry meta-analyses, which are small samples, this was not required.

### Sex-stratified analyses

Sex-stratified GWAS were performed in EUR. Seven cohorts with individual-level data available and a sample size >1,000 in both sexes were included: MVP, UKB–EUR1, UKB–EUR2, iPSYCH1, iPSYCH2, AGDS and TWINS. The same QCs and association analyses were applied as in the combined samples.

### Independent variants and conditional analyses

We identified the lead variants using PLINK with parameters of clumping region 500 kb and LD *r*^2^ = 0.1. We then ran conditional analyses using Genome-wide Complex Trait Analysis conditional and joint analysis (GCTA-COJO)^74^ to define conditionally independent variants among the lead variants using the 1000 Genomes Project phase 3 as the LD reference panel. Any two independent variants <1 Mb apart whose clumped regions overlapped were merged into one locus.

### Cross-ancestry lookup

For the 85 independent variants associated in EUR, we looked up the associations in non-EUR groups. If the variants were not observed in another ancestry, we substituted proxy SNPs defined as associated with PAU (*P* < 5 × 10^−8^) and in high LD with the EUR lead SNP (*r*^2^ ≥ 0.8).

### SNP-based heritability (*h*^2^)

SNP-based *h*^2^ for common SNPs mapped to HapMap3 was estimated in EUR, AFR and LA ancestries using LD Score regression (LDSC)^75^; corresponding populations in the 1000 Genomes Project phase 3 were used as LD reference panels. For PAU in EUR, we only estimated the observed-scale *h*^2^. For AUD, both observed-scale *h*^2^ and liability-scale *h*^2^ were estimated, using population lifetime prevalence estimates of 0.326, 0.220 and 0.229 in EUR, AFR and LA, respectively^2^. These prevalence estimates were for lifetime DSM-5 AUD in the United States, which could introduce bias given the different definitions and prevalence in different cohorts. By default, LDSC removes SNPs with sample size <90th percentile *N*/2. Here, we skipped this filtering and kept all SNPs for analyses because we did basic filtering based on the number of cohorts and sample size. The final number of SNPs in the analyses ranged from 527,994 to 1.17M.

### Cross-ancestry genetic correlation

We estimated the genetic correlations between different ancestries using Popcorn^76^, which can estimate both the genetic-effect correlation (*ρ*_ge_) as correlation coefficient of the per-allele SNP effect sizes and the genetic-impact correlation (*ρ*_gi_) as the correlation coefficient of the ancestry-specific allele variance-normalized SNP effect sizes. Populations in 1000 Genomes were used as reference for their corresponding population. A large sample size and number of SNPs are required for accurate estimation, which explains the nonrobust estimates for EAS and SAS samples.

### Within- and cross-ancestry fine mapping

We performed fine mapping using MsCAVIAR^77^, which can leverage LD information from multiple ancestries to improve fine mapping of causal variants. To reduce bias introduced by populations with small sample size, here we performed fine mapping using summary statistics from the EUR, AFR and LA populations. Three sets of analyses were conducted. The first is within-ancestry fine mapping for the 85 regions with independent variants in EUR using EUR summary data and 1000 Genomes Project phase 3 EUR LD reference data. For each region, we selected SNPs that clumped (within 500 kb and LD *r*^2^ > 0.1) with the lead SNP and with *P* < 0.05 for fine mapping. We then calculated the pair-wise LD among the selected SNPs. If two SNPs were in perfect LD (*r*^2^ = 1, indicating that they are likely to be inherited together), we randomly removed one from the analysis. The second is cross-ancestry fine mapping for the 100 regions with independent variants identified in cross-ancestry meta-analyses. For each region, we performed clumping (within 500 kb and LD *r*^2^ > 0.1) in EUR, AFR and LA summary data for the lead SNP separately, to select three sets of SNPs (*P* < 0.05) for fine mapping, with corresponding LD reference panels from the 1000 Genomes Project. For each set of SNPs, we calculated the pair-wise LD and randomly removed one SNP if *r*^2^ = 1. If the lead SNP was not presented in the EUR SNP set, we did not perform fine mapping for this region. Loci with limited numbers of variants cannot have convergent results, so they are not included in the results. After that, this cross-ancestry analysis included 92 regions. For the ten regions in which the lead SNPs are missing in both AFR and LA populations, we did within-ancestry fine mapping in EUR instead to keep the lead SNP (cross-ancestry fine mapping will only analyze the SNPs common in analyzed ancestries). Next, because the credible set length identified is related to the number of variants in the input, to provide a more direct comparison between the cross-ancestry fine mapping and the fine mapping using information only from EUR, we used the same lists of SNPs from the above 92 regions in the cross-ancestry fine mapping as for the EUR-only fine mapping. ‘Credible set’ was defined as plausible causal variants with accumulated PIP >99%. For each credible set, we report the variant with the highest PIP. We assumed that each locus contains only one causal variant by default, and increased to three at maximum if the analysis was unable to converge.

### Gene-based association analyses

We performed gene-based association analysis for PAU or AUD in multiple ancestries using MAGMA implemented in FUMA^78^. Default settings were applied. Bonferroni corrections for the number of genes tested (range from 18,390 to 19,002 in different ancestries) were used to determine GWS genes.

### TWAS

For PAU in EUR, we performed TWAS using S-PrediXcan to integrate transcriptomic data from GTEx^79^. With prior knowledge that PAU is a brain-related disorder (evidenced by significant enrichment of gene expression in several brain tissues), 13 brain tissues were analyzed. The transcriptome prediction model database and the covariance matrices of the SNPs within each gene model were downloaded from the PredictDB repository (http://predictdb.org/). Significance of the gene–tissue association was determined following Bonferroni correction for the total number of gene–tissue pairs (*P* < 0.05/166,064 = 3.01 × 10^−7^). We also used S-MultiXcan to integrate evidence across the 13 brain tissues using multivariate regression to improve association detection. In total, 18,383 genes were tested in S-MultiXcan, leading to a significance *P* value threshold of 2.72 × 10^−6^.

### Association with chromatin interactions in brain

We used H-MAGMA, a computational tool that incorporates brain chromatin interaction profiles from Hi-C, to identify risk genes associated with PAU based on EUR inputs. Six brain annotations were used: fetal brain, adult brain, adult midbrain dopaminergic, iPSC-derived astrocyte, iPSC-derived neuron and cortical neuron. In total, 319,903 gene–chromatin associations were analyzed across the six brain annotations. Significant genes were those with a *P* value below the Bonferroni corrected value for the total number of tests (*P* < 0.05/319,903 = 1.56 × 10^−7^).

### Probabilistic fine mapping of TWAS

We performed fine mapping for TWAS in EUR using FOCUS, a method that models correlation among TWAS signals to assign a PIP for every gene in the risk region to explain the observed association signal. The estimated credible set containing the causal gene can be prioritized for functional assays. FOCUS used 1000 Genomes Project EUR samples as the LD reference and multiple expression quantitative trait loci reference panel weights. Under the model of PAU as substantially a brain disorder, we did fine mapping while prioritizing predictive models using a brain tissue-prioritized approach.

### Drug repurposing

To match inferred transcriptional patterns of PAU with transcriptional patterns induced by perturbagens, we related our S-PrediXcan results to signatures from the Library of Integrated Network-based Cellular Signatures L1000 database^80^. This database catalogs in vitro gene expression profiles (signatures) from thousands of compounds from >80 human cell lines (level 5 data from phase I: GSE92742 and phase II: GSE70138). Our analyses included signatures of 829 chemical compounds in five neuronal cell lines (NEU, NPC, MNEU.E, NPC.CAS9 and NPC.TAK). To test significance of the association between PAU signatures and Library of Integrated Network-based Cellular Signatures perturbagen signatures, we followed the procedure from So et al.^81^. Briefly, we computed weighted (by proportion of heritability explained) Pearson correlations between transcriptome-wide brain associations and in vitro L1000 compound signatures using the metafor package^82^ in R. We treated each L1000 compound as a fixed effect incorporating the effect size (*r*_weighted_) and sampling variability (se^2^) from all signatures of a compound (for example, across all time points and doses). We only report those perturbagens that were associated after Bonferroni correction (*P* < 0.05/829 = 6.03 × 10^−5^).

### Cross-ancestry PRS

We used PRS-CSx, a method that couples genetic effects and LD across ancestries via a shared continuous shrinkage (CS) prior, to calculate the posterior effect sizes for SNPs mapped to HapMap3. Three sets of AUD GWAS summary data were use as input and corresponding posterior effect sizes in each ancestry were generated: EUR (without AUDIT–P from UKB, *N*_effective_ = 352,373), AFR (*N*_effective_ = 105,433) and LA (*N*_effective_ = 30,023). Three sets of AUD PRS based on the posterior effect sizes were calculated for UKB–EUR1 and UKB–EUR2 individuals using PLINK, following standardization (zero mean and unit variance) for each PRS. For each related pair (≥3rd degree, kinship coefficient ≥0.0442 as calculated by UKB), we removed the individual with the lower AUDIT–P score, or randomly if they had the same score, leaving 123,565 individuals in UKB–EUR1 and 17,401 in UKB–EUR2. Then, we ran linear regression for AUDIT–P in UKB–EUR2 as a validation dataset using PRS_EUR_, PRS_AFR_ and PRS_LA_ as independent variables. The corresponding regression coefficients were used as weights in the test dataset (UKB–EUR1) to calculate the final PRS: PRS_final_ = *ω*_EUR_ × PRS_EUR_ + *ω*_AFR_ × PRS_AFR_ + *ω*_LA_ × PRS_LA_. We used linear regression to test the association between AUDIT–P and PRS_final_ after standardization, correcting for age, sex and the first ten PCs. We also ran a null model of association between AUDIT–P and covariates only, to calculate the variance explained (*R*^2^) by PRS_final_. For comparison, we also calculated PRS in UKB–EUR1 using only the AUD summary data in EUR, then calculated the variance explained by PRS_single_. The improved PRS association was measured as the difference of the variance explained (Δ*R*^2^).

### Genetic correlation

Genetic correlations (*r*_g_) between PAU or AUD and traits of interest were estimated using LDSC. For EUR, we tested *r*_g_ between PAU and 49 traits using published summary data and the EUR LD reference from the 1000 Genomes Project. The *r*_g_ with *P* values <1.02 × 10^−3^ were considered significant. For AFR, we tested *r*_g_ between AUD and 13 published traits in AFR using MVP in-sample LD (most of the analyzed AFR were from MVP) built from 1,000 randomly selected AFR individuals by cov-LDSC^83^. The *r*_g_ with *P* values <3.85 × 10^−3^ (0.05/13) in AFR were considered as significant. For comparison, we also tested *r*_g_ using 1000 Genomes AFR as the LD reference, which showed similar estimates.

### PAU PRS for phenome-wide associations

We calculated PRS using PRS-CS for PAU (based on the EUR meta-analysis of PAU) in 131,500 individuals of EUR ancestry, and PRS for AUD (based on the AFR meta-analysis of AUD) in 27,494 individuals of AFR ancestry in four independent datasets (Vanderbilt University Medical Center’s Biobank, Mount Sinai (Bio*Me*), Mass General Brigham Biobank (MGBB)^84^ and Penn Medicine Biobank (PMBB)^85^) from the PsycheMERGE Network^86^, followed by PheWAS. Details for each dataset are described below.

### Vanderbilt University Medical Center’s Biobank

Genotyping of individuals was performed using the Illumina MEGEX array. Genotypes were filtered for SNP and individual call rates, sex discrepancies and excessive heterozygosity using PLINK. Imputation was conducted using the Michigan Imputation Server based on the Haplotype Reference Consortium reference panel. PCA using FlashPCA2 (ref. ^87^) combined with CEU, YRI and CHB reference sets from the 1000 Genomes Project phase 3 was conducted to determine participants of AFR and EUR ancestry. One individual from each pair of related individuals was removed (${\hat{\mathrm{p}}}$ > 0.2). This resulted in 12,384 AFR and 66,903 EUR individuals for analysis.

### Bio*Me*

From the Bio*Me* biobank, the Illumina Global Screening Array was used to genotype the Bio*Me* samples. The SNP-level QC removed SNPs with (1) MAF <0.0001, (2) HWE *P* value ≤1 × 10^−6^ and (3) call rate <98%. The individual-level QC removed participants with (1) sample call rate <98% and (2) heterozygosity *F* coefficient ≥3 s.d. In addition, one individual from each pair of related samples with a genomic relatedness (proportion identity by descent) >0.125 was removed (–rel-cutoff=0.125 in PLINK). Imputation was performed using 1000 Genomes phase 3 data. Each ancestry was confirmed by the genetic PC plot. A final sample size of 4,727 AFR and 9,544 EUR individuals were included for this study.

### MGBB

Individuals in the MGBB were genotyped using the Illumina Multi-Ethnic Global array with hg19 coordinates. Variant-level QC filters removed variants with a call rate <98% and those that were duplicated across batches, monomorphic, not confidently mapped to a genomic location or associated with genotyping batch. Sample-level QC filters removed individuals with a call rate less than 98%, excessive autosomal heterozygosity (±3 s.d. from the mean) or discrepant self-reported and genetically inferred sex. PCs of ancestry were calculated in the 1000 Genomes phase 3 reference panel and subsequently projected onto the MGBB dataset, where a random forest classifier was used to assign ancestral group membership for individuals with a prediction probability >90%. The Michigan Imputation Server was then used to impute missing genotypes with the Haplotype Reference Consortium dataset serving as the reference panel. Imputed genotype dosages were converted to hard-call format and subjected to further QC, where SNPs were removed if they exhibited poor imputation quality (INFO <0.8), low MAF (<1%), deviations from HWE (*P* < 1 × 10^−10^) or missingness (variant call rate <98%). Only unrelated individuals (${\hat{\mathrm{p}}}$ < 0.2) of EUR ancestry were included in the present study. These procedures yielded a final analytic sample of 25,698 individuals in the MGBB.

### PMBB

PMBB is approved under IRB protocol no. 813913. Genotyping of individuals was performed using the Illumina Global Screening Array. QC removed SNPs with marker call rate <95% and sample call rate <90%, and individuals with sex discrepancies. Imputation was performed using Eagle2 (ref. ^88^) and Minimac4 on the TOPMed Imputation Server. One individual from each pair of related individuals (${\hat{\mathrm{p}}}$ threshold of 0.25) were removed from analysis. PCA was conducted using smartpca^61^ and the HapMap3 dataset to determine genetic ancestry. This resulted in 10,383 AFR and 29,355 EUR individuals for analysis.

### PheWAS

The AFR AUD PRS and EUR PAU PRS scores in each dataset were standardized for the PheWAS analyses. ICD-9 and -10 codes were extracted from the electronic health record and mapped to phecodes. Individuals were considered cases if they had two instances of the phecode. We conducted PheWAS by fitting a logistic regression for each phecode within each biobank. Covariates included sex, age and the top ten PCs. PheWAS results were meta-analyzed within each ancestral group across biobanks (AFR 27,494 and EUR 131,500) using the PheWAS package^89^ in R. Phecodes with *N*_case_ < 100 were removed, resulting in the testing of 1,493 phenotypes in EUR and 793 in AFR. We applied a Bonferroni correction to control for multiple comparisons (*P* < 0.05/1493 = 3.35 × 10^−5^ in EUR and *P* < 0.05/793 = 6.31 × 10^−5^ in AFR).

### Yale–Penn

We also conducted PheWAS in Yale–Penn, a deeply phenotyped cohort with comprehensive psychiatric assessments (SUDs and psychiatric disorders) and assessments for physical and psychosocial traits^28^. QC and creation of the PheWAS dataset have been described previously^90^. We calculated PRS for PAU in EUR and AUD in AFR (using summary statistics that leave out the Yale–Penn 3 and PGC sample, which includes Yale–Penn 1). We conducted PheWAS by fitting logistic regression models for binary traits and linear regression models for continuous traits. We used sex, age at recruitment and the top ten genetic PCs as covariates. We applied a Bonferroni correction to control for multiple comparisons.

### Reporting summary

Further information on research design is available in the Nature Portfolio Reporting Summary linked to this article.

## Online content

Any methods, additional references, Nature Portfolio reporting summaries, source data, extended data, supplementary information, acknowledgements, peer review information; details of author contributions and competing interests; and statements of data and code availability are available at 10.1038/s41591-023-02653-5.

### Supplementary information

Supplementary Information A list of members of VA Million Veteran Program and their affiliations.

Reporting Summary

Supplementary Table Supplementary Tables 1–20.

## Data Availability

The full summary-level association data from the within-ancestry and cross-ancestry meta-analyses and sex-stratified meta-analyses in EUR ancestry are publicly available through the Gelernter Lab website without restriction (https://medicine.yale.edu/lab/gelernter/stats/) and dbGaP (accession number phs001672, under the ‘Addiction’ Analysis; registration and approval are needed following dbGaP’s data accessing process).

## Code availability

All software used in this study is publicly available. EIGENSOFT; FLASHPCA2; SHAPEIT4; Minimac4; EAGLE2; Michigan Imputation Server, https://imputationserver.sph.umich.edu/index.html#!; TOPMed Imputation Server, https://imputation.biodatacatalyst.nhlbi.nih.gov/#!; RICOPILI; PLINK; BOLT-LMM; SAIGE; GEMMA; METAL; GCTA; LDSC; cov-LDSC; Popcorn; MsCAVIAR; FUMA; S-PrediXcan and S-MultiXcan, https://github.com/hakyimlab/MetaXcan; H-MAGMA; FOCUS; PRS-CSx; PheWAS R package.
