# Supplementary material for: Multi-ancestry study of the genetics of problematic alcohol use in over 1 million individuals
Source: Nat Med. 2023 Dec 7;29(12):3184–92. doi: 10.1038/s41591-023-02653-5 (PMC10719093; doi:10.1038/s41591-023-02653-5)
Supplement: Supplementary file 1 — A list of members of VA Million Veteran Program and their affiliations. [file 41591_2023_2653_MOESM1_ESM.pdf]

nature medicine

Article

https://doi.org/10.1038/s41591-023-02653-5

Multi-ancestry study of the genetics of problematic alcohol use in over 1 million individuals

In the format provided by the authors and unedited

Supplementary Information for "Multi-ancestry study of the genetics of problematic alcohol use in over 1 million individuals"

VA Million Veteran Program Core Acknowledgement for Publications

Last updated August 3, 2021

MVP Executive Committee

- Co-Chair: J. Michael Gaziano, M.D., M.P.H. VA Boston Healthcare System
- Co-Chair: Sumitra Muralidhar, Ph.D. US Department of Veterans Affairs
- Rachel Ramoni, D.M.D., Sc.D., Chief VA Research and Development Officer US Department of Veterans Affairs
- Jean Beckham, Ph.D. Durham VA Medical Center
- Kyong-Mi Chang, M.D. Philadelphia VA Medical Center
- Philip S. Tsao, Ph.D. VA Palo Alto Health Care System
- James Breeling, M.D., Ex-Officio US Department of Veterans Affairs
- Grant Huang, Ph.D., Ex-Officio US Department of Veterans Affairs
- Juan P. Casas, M.D., Ph.D., Ex-Officio VA Boston Healthcare System

MVP Program Office

- Sumitra Muralidhar, Ph.D.
  US Department of Veterans Affairs
- Jennifer Moser, Ph.D.
  US Department of Veterans Affairs

MVP Recruitment/Enrollment

- MVP Cohort Management Director/Recruitment/Enrollment Director, Boston – Stacey B. Whitbourne, Ph.D.; Jessica V. Brewer, M.P.H.
  VA Boston Healthcare System
- VA Central Biorepository, Boston – Mary T. Brophy M.D., M.P.H.; Donald E. Humphries, Ph.D.; Luis E. Selva, Ph.D.
  VA Boston Healthcare System
- MVP Informatics, Boston – Nhan Do, M.D.; Shahpoor (Alex) Shayan, M.S.
  VA Boston Healthcare System
- MVP Data Operations/Analytics, Boston – Kelly Cho, M.P.H., Ph.D.
  VA Boston Healthcare System
- Director of Regulatory Affairs – Lori Churby, B.S.
  VA Palo Alto Health Care System
- MVP Coordinating Centers
  - Cooperative Studies Program Clinical Research Pharmacy Coordinating Center, Albuquerque – Todd Connor, Pharm.D.; Dean P. Argyres, B.S., M.S.
    New Mexico VA Health Care System

- Genomics Coordinating Center, Palo Alto – Philip S. Tsao, Ph.D.
  VA Palo Alto Health Care System
- MVP Boston Coordinating Center, Boston - J. Michael Gaziano, M.D., M.P.H.
  VA Boston Healthcare System
- MVP Information Center, Canandaigua – Brady Stephens, M.S.
  Canandaigua VA Medical Center

MVP Science

Saiju Pyarajan Ph.D.
VA Boston Healthcare System
Philip S. Tsao, Ph.D.
VA Palo Alto Health Care System

- Data Core - Kelly Cho, M.P.H, Ph.D.
  VA Boston Healthcare System
- VA Informatics and Computing Infrastructure (VINCI) – Scott L. DuVall, Ph.D.
  VA Salt Lake City Health Care System
- Data and Computational Sciences – Saiju Pyarajan, Ph.D.
  VA Boston Healthcare System
- Statistical Genetics – Elizabeth Hauser, Ph.D.
  Durham VA Medical Center
  Yan Sun, Ph.D.
  Atlanta VA Medical Center
  Hongyu Zhao, Ph.D.
  West Haven VA Medical Center

Current MVP Local Site Investigators

- Atlanta VA Medical Center (Peter Wilson, M.D.)
- Bay Pines VA Healthcare System (Rachel McArdle, Ph.D.)
- Birmingham VA Medical Center (Louis Dellitalia, M.D.)
- Central Western Massachusetts Healthcare System (Kristin Mattocks, Ph.D., M.P.H.)
- Cincinnati VA Medical Center (John Harley, M.D., Ph.D.)
- Clement J. Zablocki VA Medical Center (Jeffrey Whittle, M.D., M.P.H.)
- VA Northeast Ohio Healthcare System (Frank Jacono, M.D.)
- Durham VA Medical Center (Jean Beckham, Ph.D.)
- Edith Nourse Rogers Memorial Veterans Hospital (John Wells., Ph.D.)
- Edward Hines, Jr. VA Medical Center (Salvador Gutierrez, M.D.)
- Veterans Health Care System of the Ozarks (Kathrina Alexander, M.D.)
- Fargo VA Health Care System (Kimberly Hammer, Ph.D.)
- VA Health Care Upstate New York (James Norton, Ph.D.)
- New Mexico VA Health Care System (Gerardo Villareal, M.D.)

- VA Boston Healthcare System (Scott Kinlay, M.B.B.S., Ph.D.)
- VA Western New York Healthcare System (Junzhe Xu, M.D.)
- Ralph H. Johnson VA Medical Center (Mark Hamner, M.D.)
- Columbia VA Health Care System (Roy Mathew, M.D.)
- VA North Texas Health Care System (Sujata Bhushan, M.D.)
- Hampton VA Medical Center (Pran Iruvanti, D.O., Ph.D.)
- Richmond VA Medical Center (Michael Godschalk, M.D.)
- Iowa City VA Health Care System (Zuhair Ballas, M.D.)
- Eastern Oklahoma VA Health Care System (River Smith, Ph.D.)
- James A. Haley Veterans' Hospital (Stephen Mastorides, M.D.)
- James H. Quillen VA Medical Center (Jonathan Moorman, M.D., Ph.D.)
- John D. Dingell VA Medical Center (Saib Gappy, M.D.)
- Louisville VA Medical Center (Jon Klein, M.D., Ph.D.)
- Manchester VA Medical Center (Nora Ratcliffe, M.D.)
- Miami VA Health Care System (Ana Palacio, M.D., M.P.H.)
- Michael E. DeBakey VA Medical Center (Olaoluwa Okusaga, M.D.)
- Minneapolis VA Health Care System (Maureen Murdoch, M.D., M.P.H.)
- N. FL/S. GA Veterans Health System (Peruvemba Sriram, M.D.)
- Northport VA Medical Center (Shing Shing Yeh, Ph.D., M.D.)
- Overton Brooks VA Medical Center (Neeraj Tandon, M.D.)
- Philadelphia VA Medical Center (Darshana Jhala, M.D.)
- Phoenix VA Health Care System (Samuel Aguayo, M.D.)
- Portland VA Medical Center (David Cohen, M.D.)
- Providence VA Medical Center (Satish Sharma, M.D.)
- Richard Roudebush VA Medical Center (Suthat Liangpunsakul, M.D., M.P.H.)
- Salem VA Medical Center (Kris Ann Oursler, M.D.)
- San Francisco VA Health Care System (Mary Whooley, M.D.)
- South Texas Veterans Health Care System (Sunil Ahuja, M.D.)
- Southeast Louisiana Veterans Health Care System (Joseph Constans, Ph.D.)
- Southern Arizona VA Health Care System (Paul Meyer, M.D., Ph.D.)
- Sioux Falls VA Health Care System (Jennifer Greco, M.D.)
- St. Louis VA Health Care System (Michael Rauchman, M.D.)
- Syracuse VA Medical Center (Richard Servatius, Ph.D.)
- VA Eastern Kansas Health Care System (Melinda Gaddy, Ph.D.)
- VA Greater Los Angeles Health Care System (Agnes Wallbom, M.D., M.S.)
- VA Long Beach Healthcare System (Timothy Morgan, M.D.)
- VA Maine Healthcare System (Todd Stapley, D.O.)
- VA New York Harbor Healthcare System (Peter Liang, M.D., M.P.H.)
- VA Pacific Islands Health Care System (Daryl Fujii, Ph.D.)
- VA Palo Alto Health Care System (Philip Tsao, Ph.D.)

- VA Pittsburgh Health Care System (Patrick Strollo, Jr., M.D.)
- VA Puget Sound Health Care System (Edward Boyko, M.D.)
- VA Salt Lake City Health Care System (Jessica Walsh, M.D.)
- VA San Diego Healthcare System (Samir Gupta, M.D., M.S.C.S.)
- VA Sierra Nevada Health Care System (Mostaqul Huq, Pharm.D., Ph.D.)
- VA Southern Nevada Healthcare System (Joseph Fayad, M.D.)
- VA Tennessee Valley Healthcare System (Adriana Hung, M.D., M.P.H.)
- Washington DC VA Medical Center (Jack Lichy, M.D., Ph.D.)
- W.G. (Bill) Hefner VA Medical Center (Robin Hurley, M.D.)
- White River Junction VA Medical Center (Brooks Robey, M.D.)
- William S. Middleton Memorial Veterans Hospital (Prakash Balasubramanian, M.D.)
