# Supplementary material for: Multi-ancestry study of the genetics of problematic alcohol use in over 1 million individuals
Source: Nat Med. 2023 Dec 7;29(12):3184–92. doi: 10.1038/s41591-023-02653-5 (PMC10719093; doi:10.1038/s41591-023-02653-5)
Supplement: Supplementary file 2 — Reporting Summary [file 41591_2023_2653_MOESM2_ESM.pdf]

nature portfolio

Corresponding author(s): Hang Zhou

Last updated by author(s): Sep 28, 2023

Reporting Summary

Nature Portfolio wishes to improve the reproducibility of the work that we publish. This form provides structure for consistency and transparency in reporting. For further information on Nature Portfolio policies, see our Editorial Policies and the Editorial Policy Checklist.

Statistics

For all statistical analyses, confirm that the following items are present in the figure legend, table legend, main text, or Methods section.

n/a	Confirmed	
☐	☒	The exact sample size (*n*) for each experimental group/condition, given as a discrete number and unit of measurement
☐	☒	A statement on whether measurements were taken from distinct samples or whether the same sample was measured repeatedly
☐	☒	The statistical test(s) used AND whether they are one- or two-sided<br>*Only common tests should be described solely by name; describe more complex techniques in the Methods section.*
☐	☒	A description of all covariates tested
☐	☒	A description of any assumptions or corrections, such as tests of normality and adjustment for multiple comparisons
☐	☒	A full description of the statistical parameters including central tendency (e.g. means) or other basic estimates (e.g. regression coefficient) AND variation (e.g. standard deviation) or associated estimates of uncertainty (e.g. confidence intervals)
☐	☒	For null hypothesis testing, the test statistic (e.g. *F*, *t*, *r*) with confidence intervals, effect sizes, degrees of freedom and *P* value noted<br>*Give P values as exact values whenever suitable.*
☒	☐	For Bayesian analysis, information on the choice of priors and Markov chain Monte Carlo settings
☒	☐	For hierarchical and complex designs, identification of the appropriate level for tests and full reporting of outcomes
☐	☒	Estimates of effect sizes (e.g. Cohen's *d*, Pearson's *r*), indicating how they were calculated

Our web collection on statistics for biologists contains articles on many of the points above.

Software and code

Policy information about availability of computer code

Data collection	No custom software or algorithms were developed for data collection.
Data analysis	All software used in the analyses is described in the manuscript. All are publicly available and links or references are provided in the manuscript.<br>Principal component analysis (PCA) analyses were performed using smartpca in Eigensoft (v7.2.1) and FLASHPCA2.<br>Quality controls were performed using PLINK v1.9 and Ricopili pipeline (v2019_Oct_15.001).<br>Association analyses were performed using BOLT-LMM (v2.3.4), SAIGE (mixed-model, v1.0.0), GEMMA (v0.98) and Ricopili pipeline (v2019_Oct_15.001).<br>Meta-analyses were performed using METAL (released on 2011-03-25) with effective sample size weighting.<br>We identified the lead variants using PLINK (v1.9) with parameters of clumping region 500 kb and LD r2 0.1. We then ran conditional analyses using GCTA-COJO (v1.92.1) to define conditionally independent variants among the lead variants using the 1000 Genomes Project phase3 as the LD reference panel.<br>SNP-based heritability for common SNPs mapped to HapMap3 was estimated using LDSC (v1.0.0); corresponding populations in the 1000 Genomes Project phase3 were used as LD reference panels. Genetic correlations were estimated using LDSC.<br>The genetic correlations between different ancestries were estimated using Popcorn.<br>Fine-mapping was performed using MsCAVIAR (v1).<br>Gene-based association analysis for PAU or AUD in multiple ancestries was performed using MAGMA (v1.0.8) implemented in FUMA.<br>Transcriptome-wide association study was performed sing S-PrediXcan and S-MultiXcan to integrate transcriptomic data in 13 brain tissues from GTEx.<br>H-MAGMA (v1.0.8) incorporates brain chromatin interaction profiles from Hi-C was used to identify risk genes associated with PAU based on EUR inputs. Six brain annotations were used: fetal brain, adult brain, adult midbrain dopaminergic, iPSC-derived astrocyte, iPSC-derived

neuron and cortical neuron.
Fine-mapping for TWAS in EUR was performed using FOCUS (v0.6.10). FOCUS used 1000 Genomes Project EUR samples as the LD reference and multiple eQTL reference panel weights that include GTEx_v7.
For drug repurposing, we searched in OpenTargets.org for druggability and medication target status based on their nearest genes and we related our S-PrediXcan results to signatures from the Library of Integrated Network-based Cellular Signatures (LINCs) L1000 database.
Cross-ancestry polygenic risk score analyses were performed using PRS-CSx (released on July 29, 2021).
Phenome-wide association analyses were performed using PheWAS R package (released in 2018).

For manuscripts utilizing custom algorithms or software that are central to the research but not yet described in published literature, software must be made available to editors and reviewers. We strongly encourage code deposition in a community repository (e.g. GitHub). See the Nature Portfolio guidelines for submitting code & software for further information.

Data

Policy information about availability of data

All manuscripts must include a data availability statement. This statement should provide the following information, where applicable:

- Accession codes, unique identifiers, or web links for publicly available datasets
- A description of any restrictions on data availability
- For clinical datasets or third party data, please ensure that the statement adheres to our policy

The full summary-level association data from the within-ancestry and cross-ancestry meta-analyses and sex-stratified meta-analyses in European ancestry are publicly available through the Gelernter Lab website (https://medicine.yale.edu/lab/gelernter/stats/) and dbGaP (accession number phs001672).

Human research participants

Policy information about studies involving human research participants and Sex and Gender in Research.

Reporting on sex and gender

We conducted analyses in both sexes with sex as a covariate.
We also performed sex-stratified GWAS in EUR. Seven cohorts with individual-level data available and a sample size >1,000 in both sexes were included: MVP, UKB-EUR1, UKB-EUR2, iPSYCH1, iPSYCH2, AGDS and TWINS. The same quality controls and association analyses were applied as in the combined samples.

Population characteristics

In the MVP samples, all five ancestral groups were included (European, N=448,141; African, N=115,430; Latin American, N=38,962; East Asian, N=6,955; South Asian, N=496), with a mean age of 62 and 91.2% are males. In the UK Biobank, both European and South Asian ancestries were included, 56.4% are females. FinnGen (56.5% are females), QIMR (69.2% are females), and iPSYCH (52.8% are females) only contain European samples. PGC contains both European and African ancestries, 51.0% are females. Yale-Penn3 contains both European and African ancestries, 51.3% are females. The published East Asian cohorts have 22.4% females.

Recruitment

MVP participants were recruited through the U.S. Veterans Administration (VA) Million Veteran Program, which advertised and solicited patients receiving medical care through the VA. They gave informed consent for use of their self-report information and access to their electronic medical record. They also provided a blood sample for DNA extraction and genotyping. The MVP samples are predominantly male (>91%), which might limit the power to detect female specific loci. PGC and Yale-Penn 3 participants were recruited separately for each cohort according to their respective study designs. UK Biobank participants were recruited across the UK. The iPSYCH samples were selected from a baseline birth cohort comprising all singletons born in Denmark between May 1, 1981, and December 31, 2008. The QIMR Australian Genetics of Depression Study (AGDS) recruited >20,000 participants with major depression between 2017 and 2020. The Australian twin-family study of alcohol use disorder (TWINS, including Australian Alcohol and Nicotine Studies) participants were recruited from adult twins and their relatives who had participated in questionnaire- and interview-based studies on alcohol and nicotine use and alcohol-related events or symptoms. The Australian Genetics of Bipolar Disorder Study (GBP) recruited >5,000 participants living with bipolar disorder between 2018 and 2021.

Ethics oversight

The Central VA Institutional Review Board (IRB) and site-specific IRBs approved the MVP study. All relevant ethical regulations for work with human subjects were followed in the conduct of the study and informed consent was obtained from all participants. The iPSYCH study was approved by the Scientific Ethics Committee in the Central Denmark Region (Case No 1-10-72-287-12) and the Danish Data Protection Agency

Note that full information on the approval of the study protocol must also be provided in the manuscript.

Field-specific reporting

Please select the one below that is the best fit for your research. If you are not sure, read the appropriate sections before making your selection.

☒ Life sciences ☐ Behavioural & social sciences ☐ Ecological, evolutionary & environmental sciences

For a reference copy of the document with all sections, see nature.com/documents/nr-reporting-summary-flat.pdf

Life sciences study design

All studies must disclose on these points even when the disclosure is negative.

Sample size	We used a series of standard quality control methods to yield a total N = 1,079,947 for analysis. We have used all available samples with both genotype and phenotype data in MVP Release 4, UK Biobank, QIMR cohorts, iPSYCH 1 and 2, Yale-Penn 3, and the summary statistics from the PGC, FinnGen and East Asian cohorts. We did not do a specific power calculation.
Data exclusions	In MVP, samples with duplicates, call rates <98.5%, sex mismatches, >7 relatives, or excess heterozygosity were removed. After QC, MVP R4 data contains 658,582 participants (pre-imputation). As in our previous work, we ran principal component analysis (PCA) for the R4 data and 1000 Genome phase3 reference panels. The Euclidean distances between each MVP participant and the centers of the five reference ancestral groups were calculated using the first 10 PCs, with each participant assigned to the nearest reference ancestry. A second round of PCA within each assigned ancestral group was performed and outliers with PC scores >6 standard deviations from the mean of any of the 10 PCs were removed. Participants with at least one inpatient or two outpatient International Classification of Diseases (ICD)-9/10 codes for AUD were assigned as AUD cases, while participants with zero ICD codes for AUD were controls. Those with one outpatient diagnosis were excluded from the analysis.<br><br>UKB defined White-British (WB) participants genetically. For the non-WB individuals, we used PCA to classify them into different genetic groups as was performed for MVP. Subjects with available AUDIT-P scores were included in this study.<br><br>In iPSYCH, we generated a control group around five times as large as the case groups, and to correct for the bias introduced by high comorbidity of psychiatric disorders among cases, we included within the control group individuals with psychiatric disorders (without comorbid AUD) at a proportion equal to what was observed among the cases. More details can be found in previous studies (PMID: 28924187 and doi: https://doi.org/10.1101/2020.11.30.20237768).<br><br>QIMR Berghofer cohorts were drawn from larger batches genotyped over an extended period using several different Illumina genotyping microarrays. Participants of non-EUR ancestry (defined as >6 standard deviations from the PC1 and PC2 centroids) were excluded.<br><br>PGC samples have been published. To avoid overlap with the new QIMR Berghofer cohorts, we re-analyzed the PGC data without two Australian cohorts: Australian Alcohol and Nicotine Studies and Brisbane Longitudinal Twin Study.<br><br>Participants in Yale-Penn 3 who were not exposed to alcohol are excluded in this study.<br><br>Exclusions in FinnGen and the published East Asian cohorts can be found in literatures (PMID: 36653562 and 35094024).
Replication	We did not attempt to replicate the individual SNP association in the trans-ancestral meta-analysis duet to lack of independent data of PAU. Instead, we did look up for the 85 independent variants identified in EUR in non-EUR populations. Our results showed similarity in the genetic architecture across populations. We also performed phenome-wide polygenic risk scores analyses in four independent datasets (Vanderbilt University Medical Center's Biobank, Mount Sinai BioMe, Mass General Brigham Biobank and Penn Medicine Biobank) from the PsycheMERGE Network.
Randomization	Not applicable since this is observational study.
Blinding	Not applicable since this is observational study.

Reporting for specific materials, systems and methods

We require information from authors about some types of materials, experimental systems and methods used in many studies. Here, indicate whether each material, system or method listed is relevant to your study. If you are not sure if a list item applies to your research, read the appropriate section before selecting a response.

Materials & experimental systems

n/a	Involved in the study	
☒	☐	Antibodies
☒	☐	Eukaryotic cell lines
☒	☐	Palaeontology and archaeology
☒	☐	Animals and other organisms
☒	☐	Clinical data
☒	☐	Dual use research of concern

Methods

n/a	Involved in the study	
☒	☐	ChIP-seq
☒	☐	Flow cytometry
☒	☐	MRI-based neuroimaging
